# Reviving the Fight against Opioid Overdoses: Unleashing the Power of Metal–Organic Frameworks for Morphine Removal

**DOI:** 10.1021/acsami.5c19297

**Published:** 2025-11-30

**Authors:** Kornelia Hyjek, Klaudia Dymek, Grzegorz Kurowski, Anna Boguszewska-Czubara, Barbara Budzyńska, Jorge A. R. Navarro, Emilio Borrego-Marin, Weronika Mrozek, Justyna Grymuza, Anna Pajdak, Witold Piskorz, Paweł Śliwa, Alicja Wielgosz, Anna Stachniuk, Emilia Fornal, Przemysław J. Jodłowski

**Affiliations:** † Faculty of Chemical Engineering and Technology, 272584 Cracow University of Technology, Warszawska 24, 31-155 Kraków, Poland; ‡ Department of Medical Chemistry, 49554 Medical University of Lublin, Chodźki 4A, 20-093 Lublin, Poland; § Independent Laboratory of Behavioral Studies, Medical University of Lublin, Chodźki 4A, 20-093 Lublin, Poland; ∥ Departamento de Química Inorgánica, 16741 Universidad de Granada, Granada 18071, Spain; ⊥ Strata Mechanics Research Institute, Polish Academy of Sciences, Reymonta 27, 30-059 Kraków, Poland; # Faculty of Chemistry, Jagiellonian University in Kraków, Gronostajowa 2, 30-387 Kraków, Poland; ∇ Department of Bioanalytics, Medical University of Lublin, Jaczewskiego 8b, 20-090 Lublin, Poland; ° Lukasiewicz Research Network—Krakow Institute of Technology, Zakopiańska 73, 30-418 Kraków, Poland

**Keywords:** metal−organic frameworks, morphine, overdose, in vivo, DFT

## Abstract

The lack of suitable medical adsorbents that exhibit a quick response at the time of an overdose is challenging. In this work, we focused on morphine (MORPH) adsorbents that efficiently abate MORPH during the acute overdose and provide, in a controlled, gradual manner, MORPH antagonist—naloxone (NAL) to effectively and safely reverse overdose and its side effects by using, as sorbent, zirconium metal–organic frameworks (MOFs). Three model MOFs, including UiO-66, UiO-67, and NU-1000, were characterized in terms of their ability to adsorb MORPH as well as NAL release in water and a simulated body fluid (SBF). Single MORPH adsorption on NU-1000 was 100% in water and 90 wt % in SBF solution. At the same time, in a single NAL release in SBF from the prepared NAL@UiO-67 composite was equal to 76%. Additionally, in the simultaneous experiment of MORPH adsorption and NAL release, a designed mixture of NU-1000/NAL@UiO-67 has shown maximum adsorption/release values in SBF solution of 89 and 8%, respectively. The in vivo and in vitro experiments confirmed the exceptional effect of prepared materials on the locomotor activity of mice and the low cytotoxicity of MOFs. In vivo fluorescent imaging has confirmed that the nano-MOFs obtained are mostly accumulated in the liver when administered intravenously.

## Graphical abstract

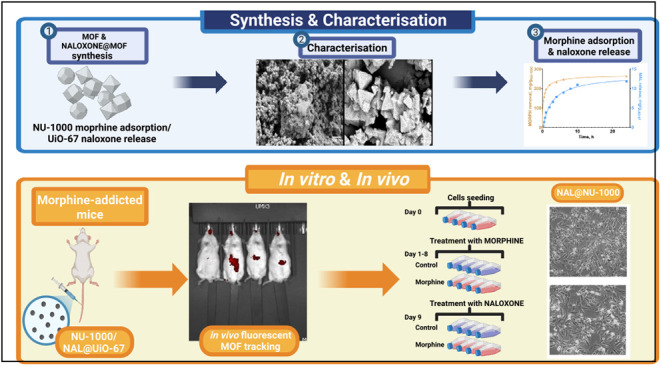

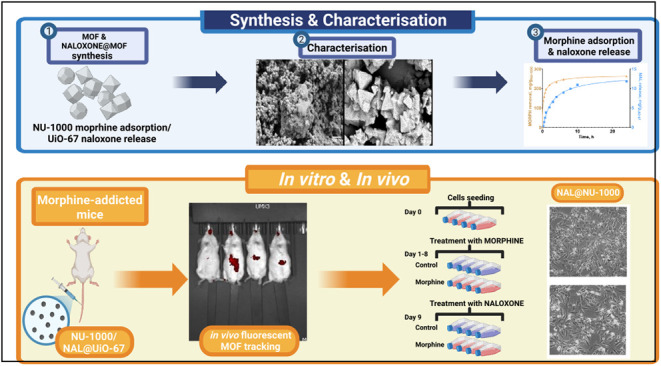

## 1 Introduction

Currently, the global use of opioid substances is increasing, especially for pain management in terminal-stage treatments. On the one hand, opioids play a crucial role in improving the quality of life of patients experiencing chronic or end-stage pain. However, despite the exceptional analgesic properties of opioids, they exhibit highly addictive side effects. A breakthrough and a stepping-stone to the forefront of medicine was the introduction of oxycodone, classified as a semisynthetic opioid. It had a faster influence on the brain, with the risk of overdose or addiction becoming much higher. As a result, the early 2000s saw the beginning of the fight against the emerging crisis, popularly referred to as the “opioid epidemic”.^1^ The success and scale of the problem are evident, for this type of addiction has been defined as a disease referred to as opioid use disorder (OUD). OUD sufferers struggle with cycles of drug use, withdrawal, and return. This behavior may be related to the effects of opioids on the central nervous system (CNS), including the reward and punishment center. Withdrawal, or overdose, of this type of compound is associated with insomnia, pain, anxiety, sweating, irritability, nausea, and respiratory problems. The disadvantageous effects of opioid withdrawal motivate the patient to take another dose, and this promotes addiction. Data from the 2018 National Survey on Drug Use and Health estimates that about 10.3 million people in the USA aged 12 and older have abused opioids, and 2 million suffer from OUD.^2^ Moreover, according to 2017 data, based on Centers for Disease Control and Prevention (CDC) statistics,^1^ nearly 48,000 Americans died from opioid overdose. It is worth noting that the specificity and potency apply to both synthetic and natural opioids. The former group, besides oxycodone (semisynthetic), includes fentanyl and carfentanyl. The specific action of morphine (MORPH), a natural painkiller, and other opioids is based on binding to μ-opioid receptors located in the brain and spinal cord. This next causes the activation of the G protein, which, consequently, initiates a molecular cascade that results in pain relief. MORPH also induces opioid respiratory depression (OIRD), a primary side effect of its use.^3^ Nonetheless, the popularity and abuse of MORPH are constantly noticed.

The opioid epidemic has forced researchers to search for compounds that could limit the side effects of opioids and combat the problem of overdose. Based on the structure, several compounds with antagonistic potency have been noted,^4^ e.g., buprenorphine, naltrexone, nalmefene, or naloxone (NAL).^4,5^ The latter is a derivative of oxymorphone. Its competitive action against μ-opioid receptors reduces the efficiency of opioids themselves.^4^ It reverses the effects of opiates, including reducing the effects of OIRD. NAL thus works both by reducing the effects of opiate intake and in dealing with overdose. It can return the patient to the state before the overdose, which is referred to as renarcalization. The popularity of NAL is also boosted by many possible forms of its administration, including nasal spray, intramuscular, intravenous, or subcutaneous injection.^6^ A very significant disadvantage in the use of NAL is its short half-life, including its short duration of action, 60–90 min, which requires frequent and repeated dosing of NAL.^4^ Without repeating the dose, OIRD will likely recur after about 40 min, while too large a dose and too rapid administration can cause cardiac arrhythmia, respiratory problems, hypertension, and even death. The compound itself is considered biocompatible and safe for humans, even in high concentrations.

The beneficial influence of NAL and its high efficacy during opioid overdose is thus irrefutable. Despite this, the short duration of action, possible side effects, and poor bioavailability after oral administration are not only a limitation for patients but also a challenge for researchers. As of today, oral administration of the compound is impossible, which stems from its weak biodistribution of 0.9 to 2%.^6^ The fast and short action of the drug can be appropriately mitigated by using specific carriers.^7^

Despite the knowledge and presence of medications that act antagonistically to MORPH, there is a lack of selective adsorbents for this type of drug on the pharmaceutical market. Currently used is activated charcoal, which, in the case of overdose, is an unsuitable and even life-threatening option for the patient^8^ Its effectiveness is also limited and dependent on the route of administration of the drug.

Among many popular and widely used substances for medical purposes, metal–organic frameworks (MOFs) seem to be reasonable and future-oriented.^9^ These modern carriers are a group of highly porous materials, combining organic and inorganic features with a unique structure, i.e., the combination of metallic centers with organic ligands.^10^ These alternative structures have found numerous potential applications from gas adsorption,^11,12^ storage,^13,14^ or catalysis,^15,16^ to drug delivery systems (DDS).^17,18^ The high potency of MOFs in the delivery of chloroquine (CQ)^19^ and acriflavine (ACF)^20^ used against SARS-CoV-2 has been studied and confirmed. Furthermore, the effect of the organometallic skeleton on extending the release of the drug and reducing the transient effects of its administration has been noted.^19,20^ MOFs have been used successfully as carriers for chemotherapeutic agents, including 5-fluorouracil (5-FU),^21^ doxorubicin (DOX),^22,23^ or cisplatin.^24,25^ These MOF grains can be coated with active compounds and serve as targeted drug delivery systems,^26,27^ or they can have shells that enhance their resistance in the human body.^28^ Over the vast majority of MOFs, zirconium-based MOFs are of great interest due to their specific properties as high specific area, pore sizes, and versatility in pre- and postmodifications, low toxicity for living organisms, and high biocompatibility.

In our recent study^29^ on the application of Zr-MOFs as an efficient adsorbent of such drugs as amphetamine, methamphetamine, MDMA, and cocaine, their superior adsorption efficiency, together with the low toxicity of the overall MOF structure and its building blocks, was confirmed via in vivo and in vitro experiments. Additionally, in our previous work,^30^ Zr-MOFs were used to release propranolol for the withdrawal of mephedrone and detoxification of human organisms. Additionally, their synthesis can be carried out in both a classical manner—solvothermal^31–33^ —and using alternative methods, such as ultrasound sonication.^34,35^ Studies of the MOFs use as sorbents have been carried out in the context of water treatment^36^ or removal of amoxicillin^37^ or mephedrone^38^ from water using MOFs.

It must also be emphasized that the literature information about the potential in vivo removal of opioids via porous materials is scarce. The literature survey on the possible application of the commonly used adsorbents is limited to the typical use of activated carbon, porous β-cyclodextrin polymers, and ferric salts (Table S1). Although in the case of activated carbon, the removal of morphine from wastewater may be as high as 50 mL L^–1^, its application as an efficient adsorbent of toxins in living organisms is rather limited due to the ill-defined structure of activated carbon itself.

Thus, this work aimed to design and characterize MOF systems that could act as a MORPH selective adsorbent and, at the same time, as an efficient NAL carrier. The adsorptive removal of MORPH and controlled release of NAL as MORPH antagonist mechanisms were thoroughly characterized via experimental and theoretical studies. Additionally, based on the in vivo studies, the potential of MOFs to mitigate MORPH-induced locomotor responses and withdrawal-related behavior induced by NAL administration was explored, suggesting their possible application in addiction treatment.

## 2 Experimental Section

### 2.1 Materials

The list of the reagents and drugs used in this study is provided in the Supporting Information File.

### 2.2 Synthesis and Characterization of Material

In our study, three different types of metal–organic frameworks—UiO-66,^20^ UiO-67,^39^ and NU-1000 ^40^ were tested. The common property of the materials used was the metallic component of the MOF, in the form of zirconium cations. The difference consisted of the ligands used, where benzene-1,4-dicarboxylic acid (ligand for UiO-66),^20^ 1,1′-biphenyl-4,4′-dicarboxylic acid (ligand for UiO-67),^39^ and an extended linker based on pyrene structure - 4,4′,4″,4‴-(pyrene-1,3,6,8-tetrayl)­tetrabenzoic acid labeled H4TBAPy (ligand in NU-1000),^40^ were adopted. The materials were synthesized according to the prescriptions available in the literature.^40^ Each of the mentioned materials was thoroughly characterized by powder X-ray diffraction analysis (PXRD), diffuse reflectance infrared Fourier transform spectroscopy (DRIFTS), low-temperature nitrogen adsorption, and Scanning Electron Microscopy (SEM). The characterization was enhanced by theoretical calculations based on density functional theory (DFT). The synthesis and characterization of pristine MOFs, along with measurement parameters and synthesis conditions, are described in detail in the Supporting Information. Composite materials obtained after NAL loading (NAL@MOF) and MORPH sorption (MORPH@MOF), described below, were characterized using the same techniques.

### 2.3 NAL Loading (NAL@MOF Composites)

The NAL@MOF composites were obtained via wet impregnation to achieve a therapeutic dose. Based on the solubility of NAL 73 mg mL^–1^ in H_2_O, 25 °C, the maximum concentration of the drug that can be introduced into 100 mg of each MOF was calculated. The obtained NAL loadings for selected MOFs are shown in Table 1.

**Table 1:** Sample Characterization Results

	MORPH sorption, mg/g_MOF_	NAL loading, mg g_MOF_^–1^	*S*_BET_, m^2^ g^–1^	*V*_NLDFT_, cm^3^ g^–1^
UiO-66			1446	0.9
MORPH@UiO-66	53		1431	0.9
NAL@UiO-66		160	893	0.5
UiO-67			1922	0.8
MORPH@UiO-67	79		11	0.006
NAL@UiO-6		150	8	0.005
NU-1000			2104	1.2
MORPH@NU-1000	134		1271	0.7
NAL@NU-1000		300	278	0.2

Before the NAL incorporation, each of the selected MOFs was activated by heating overnight under vacuum at a temperature of 120 °C for UiO-66 and NU-1000, and 80 °C for UiO-67. The resulting composite materials were subsequently denoted as NAL@MOF.

### 2.4 MORPH Sorption

MORPH sorption was carried out with different types of MOFs: UiO-66, UiO-67, and NU-1000. The sorption process was commenced by weighing 20 mg of pure, vacuum-activated MOF, which was then placed in a plastic cup. Then, 30 mL of MORPH hydrochloride (hereafter labeled MORPH) solution with an initial concentration of 0.1 mg mL^–1^ was added. Sorption was performed on a thermostatic workstation at a constant temperature of 25 °C and with continuous stirring. The kinetics of sorption were measured for 24 h, with 1 mL samples taken at the time intervals of 5, 15, 30 min, 1, 2, 3, 4, 6, and 24 h. Each sample was diluted 1.5× and then filtered through a syringe filter (Nylon, 0.22 μm). Three replicates were made for each MOF. Concentration was determined using high-pressure liquid chromatography (HPLC) with the use of a Thermo Vanquish Core HPLC System equipped with Vanquish DAD CG, Vanquish Fluorescence Detector F, Vanquish Split Sampler CT, and Column Compartment C. Separation was performed on an Accucore C18 150 × 3.0 mm^2^, 2.6 μm column. The precise conditions and parameters of the apparatus and the measurement methodology are described in detail in the Supporting Information file. We performed a MORPH sorption test on two media, distilled water and the Simulated Body Fluid (SBF). After the test time, the total solution together with the MOF was centrifuged at 6000 rpm for 10 min, leaving the obtained precipitate to air-dry. The precipitate was determined as MORPH@MOFs and used for further studies, including PXRD, DRIFT, SEM, and low-temperature N_2_ adsorption.

The study of MORPH sorption kinetics was extended to the determination of the Langmuir and the Freundlich isotherms for the material with the highest efficiency, i.e., NU-1000. For this purpose, 20 mg of each of the activated MOF was weighed, and then an aqueous MORPH solution of different concentrations, 0.1, 0.2, and 0.4 mg mL^–1^, was added. A 1 mL sample of each solution was taken before the sorption process began, and then after 6 and 24 h. Each sample was diluted 10-fold and then measured using HPLC using the method for MORPH described in SI. Subsequently, the Langmuir and the Freundlich isotherms were plotted.

### 2.5 NAL Release Test

The study of NAL release profiles was performed at a thermostatic human body temperature of 36.6 °C. For this purpose, 10 mg of NAL@MOF composite was weighed and placed in a dialysis bag (regenerated cellulose; width: 25 mm, diameter: 16 mm) of 0.02 mm wall thickness. Subsequently, 5 mL of medium was added, and the whole was placed in a plastic cup, to which 35 mL of medium and a stirrer were added. A 1 mL sample was taken at specific time intervals: 15, 30, 45 min, 1, 2, 3, 4, 6, 8, 10, and 24 h. Each time, 1 mL of the medium was refilled into the cup to equal the volume of the solution. Samples were filtered through a 0.22 μm nylon syringe filter, and then concentrations were analyzed using the HPLC (details in the SI). In all performed experiments, deionized water and SBF solution were used as the medium.

### 2.6 Simultaneous Experiment of MORPH Sorption and NAL Release

To determine the applicability of prepared materials in simultaneous MORPH adsorption and NAL controlled release, a simultaneous MORPH sorption and NAL release combined experiment was performed. In the first experiment, NU-1000 acted as a bifunctional material for the simultaneous MORPH adsorption and NAL release. In this experiment, 20 mg of previously NAL-loaded NAL@NU-1000 composite was placed in a plastic cup, and then 30 mL of 0.1 mg mL^–1^ morphine aq. solution was added. Deionized water and SBF solution were used as a medium. Collection of 1 mL samples was started at 15, 30, 45 min, 1, 2, 3, 4, 6, 8, 10, and 24 h. Each time, 1 mL of the medium was refilled into the cup to equal the volume of the solution. For those experiments, an HPLC apparatus was used. The experiment was performed in a thermostatic environment (36.6 °C). To enhance the efficacy and obtain a more gradual release profile and sorption kinetics, another experiment was performed. For this purpose, 10 mg of NAL@MOF composite material was weighed and placed in a dialysis bag (see above). A 5 mL aqueous MORPH solution with an initial concentration of 0.1 mg mL^–1^ was added to the film. It was then added to a plastic cup. In addition, 20 mg of NU-1000 material and 35 mL of aqueous MORPH solution of the same concentration were also placed in the cup. The MORPH and NAL concentration determination was determined as described in detail in the SI file.

Drug adsorption and release, and the simultaneous experiment of MORPH sorption and NAL release were evaluated in two media: distilled water and simulated body fluid (SBF, pH = 7.4). Distilled water was used to determine the intrinsic release characteristics of the formulations, while SBF was selected to mimic physiological ionic conditions according to ISO 23317:2014.^41,42^ The use of these media is consistent with pharmacopeial recommendations (Ph. Eur. 2.9.3)^42^ to reflect both idealized and physiologically relevant environments for in vitro release testing of MOF-based systems.

### 2.7 DFT Molecular Modeling and Molecular Dynamics

Initially, the position of adsorbate molecules in the MOF frameworks was roughly determined by the Monte-Carlo (MC) simulations (Metropolis algorithm, Universal Force Field,^43^ *T* = 298 K). The positions were subsequently refined at the DFT periodic boundary conditions level of theory using the VASP code.^44^ The details of methodology (e.g., the XC functional, the convergence criteria and accelerator, and the solvent modeling) are given elsewhere.^45^ The model unit cell stoichiometries and dimensions (lengths of cell vectors in Å and cell angles in degrees) were as follows. For NU-1000: C_264_H_180_O_96_Zr_18_, 39.970, 39.970, 16.580, 90.0, 90.0, 120.0; for UiO-67: C_84_H_52_O_32_Zr_6_, 19.012, 19.012, 19.012, 60.0, 60.0, 60.0; and for UiO-66: C_192_H_96_O_128_Zr_24_, 20.747, 20.747, 20.747, 90.0, 90.0, 90.0. For all unit cells, the P1 symmetry was assumed.

The adsorption energies, accounting for the basis set incompleteness, together with the charges (two population analyses: Bader and DDEC6) and bond orders (DDEC6), are summarized in Table 2. The equilibrium loading was estimated using MC simulations, and the isotherms were modeled for fugacity up to 100 kPa. The rigid host approximation was used, which can rationalize the underestimation of the modeled loading for the MOF structures with tight channels.

**Table 2:** Adsorption Energies of Organic Adsorbate in the MOF NU-1000 and UiO-67 Frameworks^a^

adsorbate molecule	*E*_ads_/eV	B.O. (ads.-MOF)	*q*_DDEC_(ads.)	*q*_Bader_(ads.)
NU-1000				
MORPH, narrow channel	–1.435	1.2471	–0.055163	–0.0406
MORPH, wide channel	–1.695	1.8979	–0.071103	–0.0446
NAL, narrow channel	–1.911	1.0449	0.015038	–0.0156
NAL, wide channel	–1.485	0.6109	–0.003604	–0.0181
NAL + MORPH, wide channel	–3.300	2.6497	0.030697	0.0000
UiO-67				
MORPH, str. 1	–1.687	1.3576	–0.051714	–0.0325
MORPH, str. 2	–0.996	0.8513	0.069087	0.0239
MORPH str. 3	–1.392	1.1246	–0.041201	–0.0303
NAL, str. 1	–1.121	0.8567	–0.007273	–0.0161
NAL, str. 2	–0.964	0.8938	0.068791	0.0148

a The total bond order, B.O. (ads.-MOF), between the adsorbed molecule/molecules and the host MOF framework is determined by employing the DDEC6 bond analysis. The accumulated charge on the adsorbate molecule/molecules, *q*_DDEC_(ads.) and *q*_Bader_(ads.) were calculated via the DDEC6 and the Bader partial charge analyses, respectively, and expressed in |e| units.

The DFT-preoptimized supercell (3 × 3 × 3) structure of NU-1000 with randomly placed MORPH, NAL, or both molecules was further used as the initial configuration for molecular dynamics simulations. The MD simulation was performed using the Gromacs 2024.3 package,^46,47^ under constant temperature, constant volume, and periodic boundary conditions.

The topology of complexes was generated by the OBGMX tool^48^­[52] using the universal force field (UFF).^43^ The atomic charges for complexes were assigned using the CHelpG calculation scheme at the HF/LanL2MB level of theory with Gaussian 16. The simulation boxes were then filled with the TIP3P water model described by the UFF force field. During the MD simulation, all involved hydrogen bonds were constrained by the LINCS algorithm with an integration step size of 1 fs. Electrostatic interactions were calculated using the particle-mesh Ewald (PME) method. The nonbonding interaction cutoff was set to 12 Å and updated every 10 steps. The simulated temperature was controlled to 300 K using the temperature coupling by velocity rescaling with a stochastic term (V-rescale). First, the energy minimization was carried out using the steepest descent method to eliminate too close contacts between atoms; then, the NVT equilibrium simulation was performed at 300 K for 100 ps; finally, a 100-ns NPT production run with isotropic pressure coupling using the Parrinello-Rahman barostat at 1 bar was performed. The visualization and analysis of the simulation results were done using the Gromacs embedded program and VMD.^49^

### 2.8 In Vitro

The H9c2(2–1) cells (ATCC-CRL-1446: Heart, Myocardium; Rat *Rattus norvegicus*) for cardiotoxicity tests, HT22 cells (mouse hippocampal neuronal cell line, CVCL_0321) for neurotoxicity tests, and HepG2 cells (ATCC-HB-8065^TM^: Human hepatoma) for hepatotoxicity tests were cultivated in complete DMEM or EMEM (for HepG2) culture medium supplemented with 10% fetal bovine serum (FBS) and 1% streptomycin/penicillin. The cells were maintained at 37 °C in a humidified incubator with a 5% CO_2_ atmosphere.

For experimental treatments, cells were seeded into eight 25 cm^2^ culture flasks in complete culture medium and incubated for 24 h to allow attachment and stabilization.

On day 1, the medium in four flasks was replaced with medium containing 10 mg mL^–1^ of MORPH, while the other four flasks received fresh complete medium only (controls). The medium was changed daily. For the MORPH-treated cells, the concentration was gradually increased each day to 15, 20, 25, 30, 35, 40, and, finally, 50 mg mL^–1^. Control cells received fresh complete medium without MORPH each time.

On day 8, cells from all flasks were treated with either free NAL (NAL)—four flasks total (2 controls and 2 MORPH-treated)—or NAL-loaded NU-1000 metal–organic framework (NAL@NU-1000)—four flasks total (2 controls and 2 MORPH-treated). The cells were then incubated under the same standard conditions for 24 h.

Starting from day 9, images of all cells were taken to visualize the effects of MORPH intoxication and NAL detoxification.

Cell viability analysis was performed based on image analysis using the ImageJ software, expressed as the percentage of confluence. For each treatment, three images were analyzed from each of two flasks.

### 2.9 In Vivo

#### 2.9.1 Zebrafish Study

All experiments were carried out at Medical University of Lublin (Poland) under standardized laboratory conditions. The fish were kept at a stable temperature of 28.5 °C with a controlled photoperiod consisting of 14 h of light followed by 10 h of darkness. Fertilized eggs were collected via natural mating. Embryos were cultured in E3 medium (pH = 7.1–7.3) composed of 17.4 μM NaCl, 0.21 μM KCl, 0.12 μM MgSO_4_, and 0.18 μM Ca­(NO_3_)_2_, and incubated at 28.5 °C. Larvae were euthanized by immersion in a 15 μM tricaine solution. Detailed methodological procedures have been previously described. All experimental procedures complied with the National Institutes of Health Guidelines for the Care and Use of Laboratory Animals and adhered to the European Directive 2010/63/EU of September 22, 2010. Ethical approval was not required for experiments involving zebrafish larvae up to 120 h postfertilization, in accordance with current regulatory standards.

#### 2.9.2 Heart Rate

Within 90 min postfertilization, embryos were carefully screened under a light microscope (Stemi 508, Zeiss) to assess viability and fertilization status. Viable embryos were then transferred—within 3 h postfertilization (hpf)—to individual wells of 96-well plates. The doses of MOFs used in the experiments were based on MORPH 25 mM absorption and were as follows: NU-1000 (2.5 mg mL^–1^), UiO-66, and UiO-67 (both 4 mg mL^–1^). Each well contained 200 μL of either a test compound MORPH (25 mM), MOFs, or a control solution, with one embryo allocated per well. The embryos remained in their respective solutions for a continuous 96 h exposure period.

At 96 hpf, larvae were allowed to acclimate at room temperature for 30 min. Following this, their heart rate was assessed using a stereomicroscope. Heartbeats were counted over a 15-s interval and multiplied by four to calculate beats per minute (bpm). For each treatment condition, 10 larvae were analyzed.

#### 2.9.3 Locomotor Activity

For evaluation of locomotor activity, the assay was performed in 5 dpf larvae, after the HB calculation, with one larva in each well of a 96 multiwell plate. EthoVision XT video tracking software (Noldus) was used for evaluating locomotor activity. The distance moved in 10 min was calculated in a light condition. For the experiment, 12 larvae were pretreated.

#### 2.9.4 Rodent Study

The experiments were conducted on male Swiss mice weighing 20–30 g. The animals were housed at a room temperature of 22 ± 1 °C with a natural day-night cycle. They had free access to standard food (Murigran pellets, Bacutil, Motycz) and tap water. After one week of adaptation and handling, the animals were divided into groups of 8–10 mice per group and prepared for testing.

All behavioral experiments were conducted between 8 A.M. and 2 P.M. Injections were administered at 8 A.M. and 6 P.M. The study was carried out in accordance with the National Institute of Health Guidelines for the Care and Use of Laboratory Animals and the European Community Council Directive for the Care and Use of Laboratory Animals. The protocol was approved by the Local Ethics Committee of the Medical University of Lublin (Committee on the Use and Care of Animals, Approval No 54/2024).

For the locomotor activity test, drugs were dissolved in *aqua pro iniectione* and administered intravenously (i.v.) at a volume of 5 mL kg^–1^. The doses of MOFs used in the experiments were based on MORPH 0.025 mg kg^–1^ absorption and were as follows: UiO-66 at 1.85 mg kg^–1^, UiO-67 at 1.55 mg kg^–1^, and NU-1000 at 0.4 mg kg^–1^. Control animals received the same volume of *aqua pro iniectione* at the respective times before testing.

To evaluate the effects of NAL@MOF on MORPH-withdrawal syndrome, the methodology was based on the study by Listos and co-workers.^50^ The doses of NAL@MOF used in the experiments were based on the calculation of release of 2 mg NAL from MOFs and were as follows: UiO-66—30 mg kg^–1^, UiO-67—30 mg kg^–1^, and NU-1000—20 mg kg^–1^. All drugs were administered intraperitoneally (i.p.) at a volume of 10 mL kg^–1^ in *aqua pro iniectione*. NAL was injected at a dose of 2 mg kg^–1^. Control animals received the same volume of saline at the respective times before testing.

### 2.10 Procedures and Tests

#### 2.10.1 Spontaneous Locomotor Activity

Spontaneous locomotor activity was evaluated using the Opto-Varimex-4 Auto-Track system (Columbus Instruments, Columbus, OH, USA). The device consists of four transparent cages with lids (43 × 43 × 32 cm^3^), each equipped with a set of four infrared emitters (each emitter has 16 laser beams) and detectors that monitor animal movements. Each mouse was placed individually into a cage for 30 min. Animals were randomly allocated into 8 groups to receive intravenous (i.v.) injections of *aqua pro iniectione*, MORPH (0.025 mg kg^–1^ or 0.05 mg kg^–1^), NU-1000 (0.04 mg kg^–1^), UiO-67 (1.55 mg kg^–1^), UiO-66 (1.85 mg kg^–1^), or MORPH coadministered with MOFs.

MORPH was administered 15 min before the MOFs, and immediately afterward, the mice were placed in the locomotor cages. Locomotor activity was measured for 30 min. The doses of MORPH were selected based on a pilot study (see Supporting Information).

Immediately after the locomotor activity test, mice were decapitated, and the whole brain tissues were collected and stored at −80 °C until further LC-MS/MS and biochemical analyses. Samples were mechanically homogenized (three steel beads) in acetonitrile (1:4 w/v). Homogenate was centrifuged at 13,000 rpm, 10 min, at +4 °C. 50 μL of supernatant was spiked with 5 μL of IS solution (25 ng mL^–1^ of psoralen). A 10 μL of aliquot was injected into the LC-MS/MS system for analysis.

#### 2.10.2 Physical Dependence and Effects of MOFs on the Expression of MORPH Withdrawal Signs in Mice

According to a previous study by Listos et al.,^50^ a state of MORPH dependence in mice was obtained by administering increasing doses of MORPH for eight consecutive days (10, 15, 20, 25, 30, 35, 40, and 50 mg kg^–1^, i.p.), twice a day. In our study, on the ninth day of the experiment, MORPH was administered at a dose of 50 mg kg^–1^ in the morning. One hour later, to induce MORPH withdrawal signs, an opioid receptor antagonist, NAL, was injected (2 mg kg^–1^, i.p.). Then, the animals were immediately placed in glass cylinders, and the number of jumps (considered as a typical sign of MORPH withdrawal) was recorded for 30 min. Control animals received *aqua pro iniectione* instead of MORPH at the respective times during the experiment.

To assess the effect of NAL@MOF on the expression of MORPH withdrawal signs, composites were administered 30 min after the last MORPH injection on day 9. The test was performed 30 min after NAL@MOF injection. Prior both i.v. and i.p. injection MOFs or NAL@MOF were dissolved in *aqua pro iniectione* and sonicated for 15 min at 36 °C to ensure proper dispersion and solubility, as well as compatibility and safety for intravenous administration.

### 2.11 LC-MS/MS Measurements and Quantification of MORPH

#### 2.11.1 Materials

Acetonitrile and methanol (both Optima LC-MS grade) were purchased from Thermo Fisher Scientific (Waltham, MA, USA). Formic acid (LC-MS grade) and certified MORPH solution of 1 mg/mL in methanol were obtained from Merck KGaA (Darmstadt, Germany). Working standard solutions of the analyte were prepared daily by dilution with methanol. Four mm Titan syringe filters (0.20 μm) from Thermo Fisher Scientific (Waltham, MA, USA). The Millipore Direct Q 3UV water purification system (Millipore, U.K.) was used to produce ultrapure water. All other chemicals were purchased from Sigma-Aldrich in the best available purity grade.

#### 2.11.2 Sample Preparation

The extraction of MORPH was performed by adding 300 μL of a cold (−20 °C) mixture of methanol and ethanol (1:1 v/v) to 100 μL of plasma. The samples were vortexed for 30 s and placed for 15 min at −20 °C. Then, samples were centrifugation at 16,000 *g* for 20 min at 4 °C. The supernatant was collected and diluted with 0.1% formic acid (1:1, v/v) and filtered through a Titan syringe filter (0.2 μm) into an autosampler vial. For the preparation of brain samples, 100 mg of the sample was homogenized with 500 μL of a cold (−20 °C) mixture of methanol and ethanol (1:1 v/v) with a laboratory ball homogenizer (Bead Mill MAX, VWR, part of Avantor, Radnor, PA, USA) using 2.8 mm diameter ceramic bead balls at 6 m/s in one 30 s cycle. The homogenates were placed at −20 °C for 12 h (overnight) and then centrifuged at 16,000 *g* for 20 min at 4 °C. The supernatant was diluted with 0.1% formic acid (1:1, v/v) and filtered through a Titan syringe filter (0.2 μm) into an autosampler vial. The obtained extracts were subjected to UHPLC-MS/MS analysis. Quantitative analysis was performed using the calibration curve prepared by fortifying control samples, which were checked to be free of analyte beforehand, with MORPH standard. Concentration ranges were selected based on therapeutic drug concentrations and were in the range of 1–50 ng mL^–1^ and 1–10 ng mL^–1^ for plasma and brain, respectively.

#### 2.11.3 LC/MS Analysis

Liquid chromatography mass spectrometry (LC/MS) analysis was performed using an Agilent Technology 1290 Infinity II series liquid chromatograph, composed of a binary pump (G7120A), a multisampler (G7167B), and a multicolumn thermostat (G7116B), coupled to an Agilent Technologies triple quadrupole mass spectrometer 6475 TQ LC/MS equipped with a JetStream Technology electrospray ion source (Agilent Technologies, Santa-Clara, CA, USA). A Zorbax Eclipse Plus C18 RRHT column (2.1 mm × 100 mm, 1.8 μm) with a Zorbax Eclipse Plus C18 (2.1 mm × 5 mm, 1.8 μm) guard column (both from Agilent Technologies) was used for chromatographic separation. The mobile phases were 0.1% formic acid in water (A) and 0.1% formic acid in acetonitrile (B). The gradient program started with 2% B, which was increased to 20% over 2 min and then to 95% over 0.1 min. Mobile phase B was held at 95% B for 3 min and then 2 min postrun at 2% B. The injection volume was 5 μL. The column temperature was maintained at 40 °C. MORPH eluted at 1.45 min. The mass spectrometer was operated in positive electrospray ionization mode (ESI+) with the following settings: ion source gas temperature, 300 °C; ion source gas flow rate, 12 L min^–1^; nebulizer pressure, 40 psi; sheath gas temperature, 350 °C; sheath gas flow rate, 12 L min^–1^; and capillary voltage, 4000 V. Fragmentor was set to 160 V. Data were acquired in multiple reaction monitoring (MRM) mode. Three MRM transmissions were monitored: 286.2 to 201 at the collision energy of 29, and 165 and 152.1 at the collision energy of 49. Agilent Mass Hunter software versions B.09.00 and B.10.02 were used for data acquisition and data qualitative analysis, respectively.

### 2.12 Statistical Analysis

The statistical analysis of the drug adsorption and release data obtained in this study was carried out using GraphPad Prism 10.6.1 software. The standard error of the mean (SEM) was calculated based on triplicate measurements and is displayed as error bars in all kinetic plots. In some cases, the error bars were smaller than the symbol size and remained within the range of experimental uncertainty. The in vitro and in vivo results were statistically calculated using *t*-test or one- or two-way ANOVA, using GraphPad Software 8.3.0. Comparisons between groups were made by employing the posthoc test (Tukey’s or Bonferroni’s test). Significant statistical differences were assumed when *p* < 0.05. GraphPad Prism version 8.00 for Windows, GraphPad Software, San Diego, CA, USA, was used. In the experiments, the following parameters were considered: (1) The results obtained in the Spontaneous locomotor activity test were presented as an arithmetic average distance (expressed in cm) traveled by a mouse ± SEM for each experimental group. (2) the average amount of jumping behavior ± standard error of measurement in the experiment assessing MORPH withdrawal intensity.

## 3 Results and Discussion

### 3.1 Characterization

In this work, a set of Zr-MOFs, namely, UiO-66, UiO-67, and NU-1000, was used as modern adsorbents of MORPH and NAL release in the treatment of acute drug overdose. The materials share the same hexanuclear Zr metal cluster but diverge in the organic linker, and consequently also in crystallinity, pore size, specific surface area, as well as surface morphology and topology.^51,52^ The effective sizes of both MORPH and NAL molecules, as well as effective pore sizes in model NU-1000 and UiO-67 used as adsorbent, were depicted in Figure 1.

**Figure 1:**
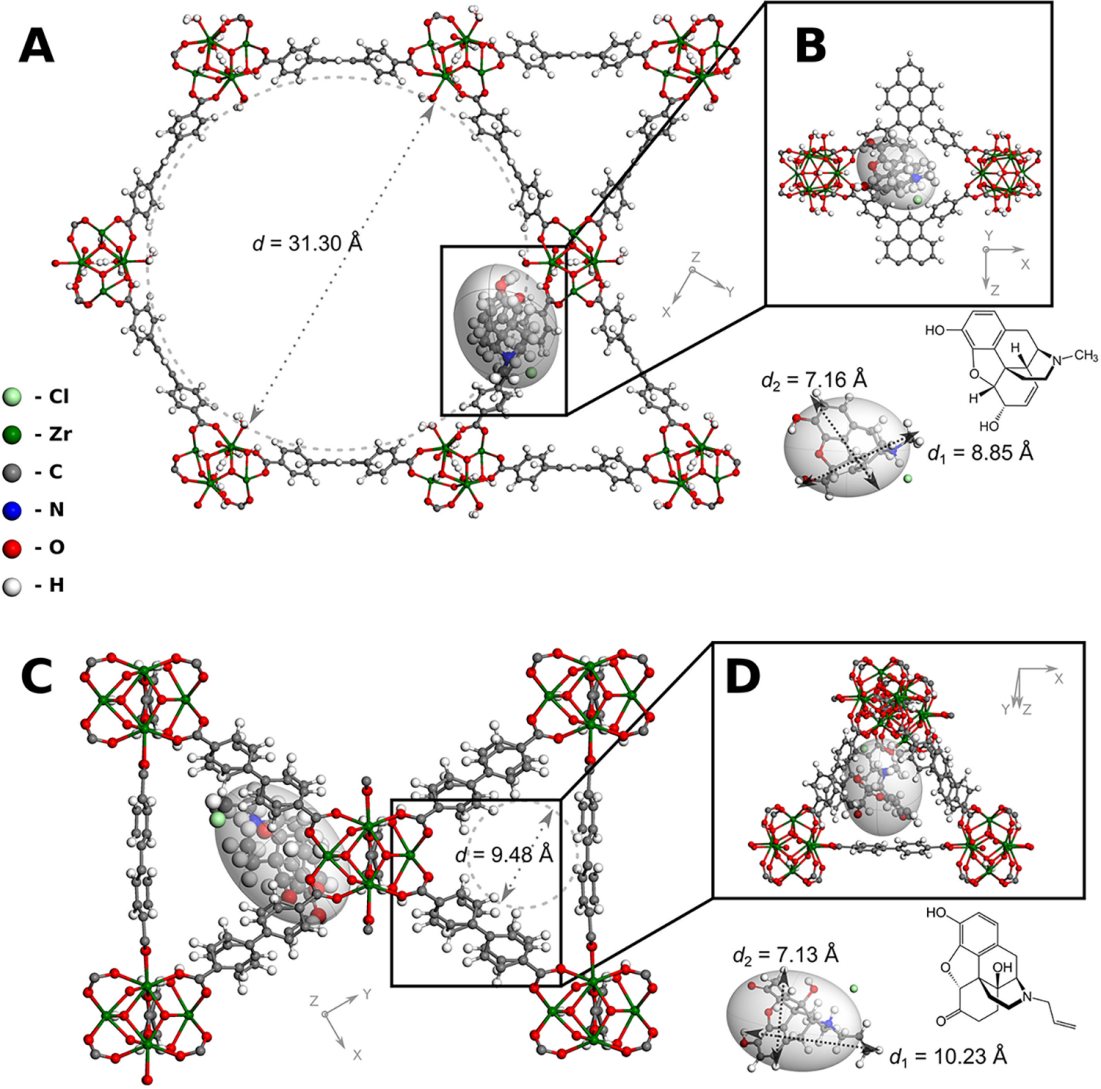
(A, B) MORPH@NU-1000; (C, D) NAL@UiO-67: (A, C) general view with approximate channels breadth; (B, D) detailed view. The cage sizes calculated with the ’nudge-and-bulge’ algorithm for finding the Maximum Inscribed Sphere are 31.70 Å and 12.02 Å for the wide channel in NU-1000 and the channel in UiO-67, respectively.

The physicochemical differences in the networks are reflected in their sorption capacities, as confirmed and explained later in the article. For example, UiO-66 is characterized by tetragonal and octahedral cages, and the addition of hydrochloric acid during its synthesis ensures the formation of additional structural defects, such as a missing cluster or a missing ligand.^53^ In the NU-1000 network with an extended ligand based on the pyrene structure, the pores are triangular and hexagonal.^54^ High crystallinity of MOF materials was confirmed by PXRD (Figures 2 and S1).

**Figure 2:**
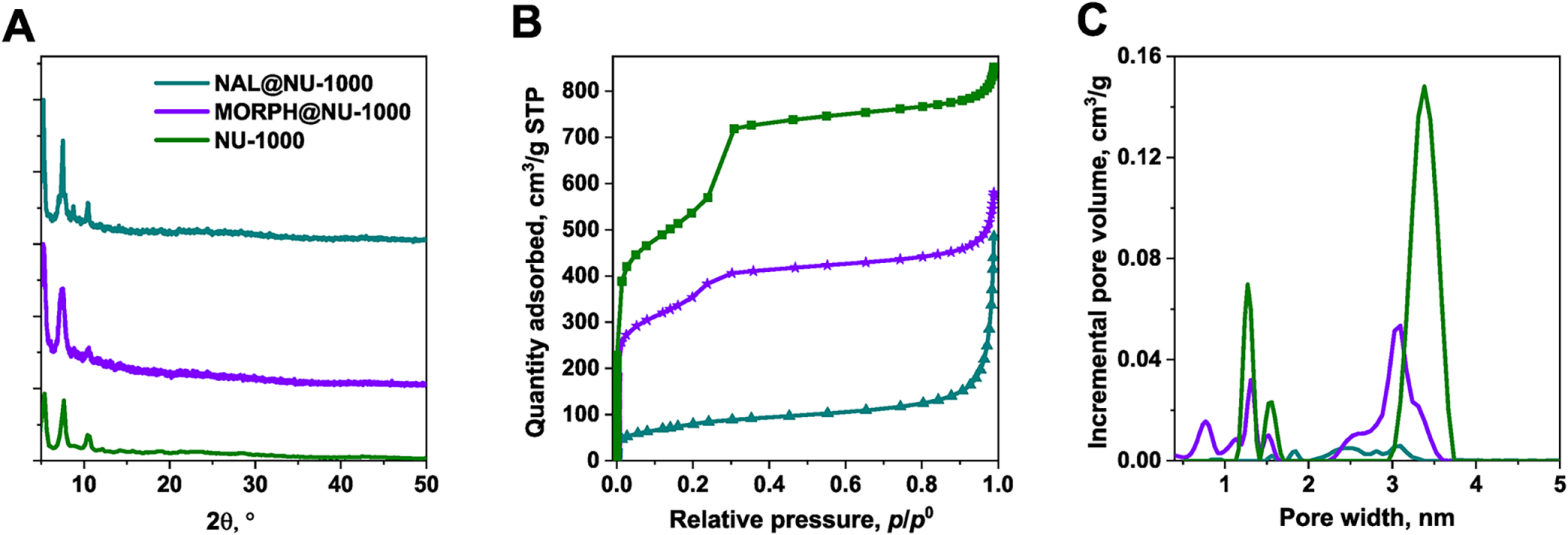
Characterization results of NU-1000 and prepared composites MORPH@NU-1000 and NAL@NU-1000; (A) PXRD; (B) N_2_ adsorption isotherms; (C) NLDFT pore size distribution.

Diffractograms for pristine UiO-66, UiO-67, and their composites are shown in Figure S1 and are consistent with data available in the literature.^16,30,55,56^ The PXRD studies performed for MORPH@MOF composites obtained after sorption of MORPH from SBF solution show loss of crystalline character and collapse of the structure for MORPH@UiO-67. Soaking the MOFs and their prolonged contact with the ions contained in the SBF affect the structure of the materials, including the formation of a layer of hydroxyapatite (HA) on the surface of MOFs. We can confirm this phenomenon by the appearance of an additional peak on the diffractogram at the 2Θ position of about 31°.^57,58^ This peak is not visible on the diffractogram of MORPH@UiO-66 and MORPH@NU-1000. In this case, the SBF solution does not affect the crystal structure of UiO-66, as well as NU-1000. The PXRD diffractograms of pristine NU-1000 and NU-1000 composites are shown in Figure 2A. In the case of MOF composites loaded with NAL, NAL@NU-1000 also presents stability, and the crystalline character of the sample is preserved. A similar situation applies to NAL@UiO-66, whose crystalline character was preserved after the drug loading process. The NAL@UiO-67 composite is unstable, and the sample does not exhibit good crystallinity, as reflected by the absence of specific peaks on the diffractogram. A similar relationship regarding the UiO-67 lattice-based composite and its lack of stability was observed by our team in an earlier study (Figure S1A).

To determine the specific surface area of the materials, as well as the pore volume, low-temperature adsorption of nitrogen was conducted (Figures 2B,C, S1B,C and Table 1). It was performed for MOFs before and after the MORPH sorption process, which helped to observe changes in surface area sizes and confirm MORPH adsorption on metal–organic frameworks. Referring to pure MOFs, the synthesized materials are characterized by different S_BET_ surface area sizes ranging from ca. 1400 m^2^ g^–1^ to as high as 2100 m^2^ g^–1^. Of which, considering the increasing specific surface areas occur successively UiO-66, UiO-67, and NU-1000. Analogous relations hold in the case of the total volume of micropores (NLDFT), where the values are between 0.8 cm^3^ g^–1^ and 1.2 cm^3^ g^–1^. The study confirms the high specific surface area values of the materials used. This also indirectly indicates the high sorption potential of the MOFs used. Measurements were also made for MORPH@MOF composites, which were obtained after 24 h of sorption of the psychoactive substance. The following changes were observed. For each composite, the specific surface area of *S*_BET_ decreases. The largest decrease was observed for MORPH@UiO-67, where the mentioned value decreased to about 11 m^2^ g^–1^. This may be due to the collapse of the UiO-67 structure in the presence of compact ions in the SBF solution, as demonstrated in our previous work. In addition, the NLDFT value drops to 0.006 cm^3^ g^–1^, which is about 2 orders of magnitude lower. The experiment emphasizes the loss of crystallinity of the material. For MORPH@UiO-66 and MORPH@NU-1000, these parameters also decreased. However, the decreased values are not as significant. It may be due to the adsorption of the drug on the MOFs while maintaining the crystalline nature of the MORPH@UiO-66 and MORPH@NU-1000. In both cases, the NLDFT values are in the range of 0.7–0.9 cm^3^ g^–1^. On the other hand, the S_BET_ value clearly decreased for MORPH@NU-1000, which may indicate a high sorption capacity and occupation of a larger part of the specific surface area by the adsorbed MORPH. There is a much smaller decrease for MORPH@UiO-66, which may emphasize the much lower sorption capacity, as confirmed later in the manuscript. In addition, a significant decrease in S_BET_ specific surface area is observable for composites loaded with NAL:NAL@MOF. The largest decrease in the value of specific surface area is noticeable for NAL@UiO-67. The BET surface area of the composite after the introduction of the antidote is 8 m^2^ g^–1^. Such a small value of the mentioned parameter is related to the collapse of the NAL@UiO-67 structure. It is due to the instability of the material. The introduction of NAL by the first moisture method into the structure of UiO-67 causes a loss of stability. Above that, the NLDFT value is 0.005 cm^3^ g^–1^. An analogous situation is observable for MOPRH@UiO-67, as described above. A significant decrease in the value of the specific surface area of S_BET_ is also evident for NAL@NU-1000. In this case, it is not due to the loss of stability of the material but is related to the loading of a large amount of NAL, amounting to 300 mg g^–1^ of NU-1000. We also observe a reduction in the pore volume of the composite to 0.2 cm^3^ g^–1^. In the case of NAL@UiO-66, a decrease in both S_BET_ and V_NLDFT_ is observable, but it is not so significant. The value of the specific surface area of the composite is 893 m^2^ g^–1^, and the pore volume is 0.5 cm^3^/g. The results of the conducted experiment are consistent with the diffractograms of the composite materials and are illustrated in Figure S1B,C. The N_2_ adsorption and pore size distribution for NU-1000, MORPH@NU-1000, and NAL@NU-1000 are shown in Figure 2B,C. It was performed for MOFs before and after the MORPH sorption process, which helped to observe changes in surface area sizes and confirm MORPH adsorption on metal–organic frameworks. Referring to pure MOFs, the synthesized materials are characterized by different S_BET_ surface area sizes ranging from ca. 1400 m^2^ g^–1^ to as high as 2100 m^2^ g^–1^. Of which, considering the increasing specific surface areas occur successively UiO-66, UiO-67, and NU-1000. Analogous relations hold in the case of the total volume of micropores (NLDFT), where the values are between 0.8 cm^3^ g^–1^ and 1.2 cm^3^ g^–1^. The study confirms the high specific surface area values of the materials used. This also indirectly indicates the high sorption potential of the MOFs used. Measurements were also made for MORPH@MOF composites, which were obtained after 24 h of sorption of the psychoactive substance. The following changes were observed. For each composite, the specific surface area of *S*_BET_ decreases. The largest decrease was observed for MORPH@UiO-67, where the mentioned value decreased to about 11 m^2^ g^–1^. This may be due to the collapse of the UiO-67 structure in the presence of compact ions in the SBF solution, as demonstrated in our previous work. In addition, the NLDFT value drops to 0.006 cm^3^ g^–1^, which is about 2 orders of magnitude lower. The experiment emphasizes the loss of crystallinity of the material. For MORPH@UiO-66 and MORPH@NU-1000, these parameters also decreased. However, the decreased values are not as significant. It may be due to the adsorption of the drug on the MOFs while maintaining the crystalline nature of the MORPH@UiO-66 and MORPH@NU-1000. In both cases, the NLDFT values are in the range of 0.7–0.9 cm^3^ g^–1^. On the other hand, the S_BET_ value clearly decreased for MORPH@NU-1000, which may indicate a high sorption capacity and occupation of a larger part of the specific surface area by the adsorbed MORPH. There is a much smaller decrease for MORPH@UiO-66, which may emphasize the much lower sorption capacity, as confirmed later in the manuscript. In addition, a significant decrease in S_BET_ specific surface area is observable for composites loaded with NAL NAL@MOF. The largest decrease in the value of specific surface area is noticeable for NAL@UiO-67. The BET surface area of the composite after the introduction of the antidote is 8 m^2^ g^–1^. Such a small value of the mentioned parameter is related to the collapse of the NAL@UiO-67 structure. It is due to the instability of the material. The introduction of NAL by the first moisture method into the structure of UiO-67 causes a loss of stability. Above that, the NLDFT value is 0.005 cm^3^ g^–1^. An analogous situation is observable for MOPRH@UiO-67, as described above. A significant decrease in the value of the specific surface area of S_BET_ is also evident for NAL@NU-1000. In this case, it is not due to the loss of stability of the material but is related to the loading of a large amount of NAL, amounting to 300 mg g^–1^ of NU-1000. We also observe a reduction in the pore volume of the composite to 0.2 cm^3^ g^–1^. In the case of NAL@UiO-66, a decrease in both *S*_BET_ and *V*_NLDFT_ is observable, but it is not so significant. The value of the specific surface area of the composite is 893 m^2^ g^–1^, and the pore volume is 0.5 cm^3^/g. The results of the conducted experiment are consistent with the diffractograms of the composite materials and are illustrated in Figure S1B,C. The N_2_ adsorption and pore size distribution for NU-1000, MORPH@NU-1000, and NAL@NU-1000 are shown in Figure 2B,C.

The SEM scanning electron microscopy was performed for the obtained materials and composites. The resulting micrographs, as well as the discussion, are provided in the SI file (Figure S2).

To determine the molecular structure of prepared materials, DRIFT spectroscopy analyses were performed for MORPH@MOF and NAL@MOF composites (Figure 3).

**Figure 3:**
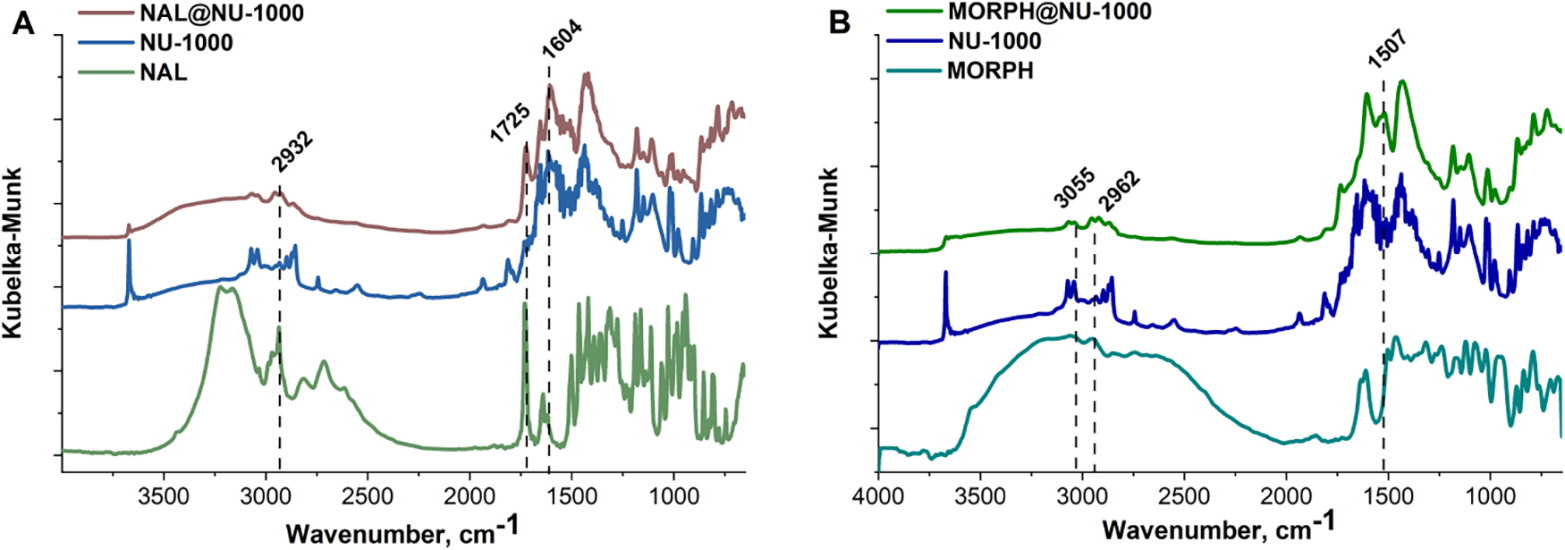
DRIFT spectra of prepared composites; (A) NAL@NU-1000; (B) MORPH@NU-1000.

A comparative analysis of the spectrum of pure MORPH and pristine MOFs (UiO-66, UiO-67, and NU-1000) with the spectra of MORPH@MOF composites was performed and shown in Figures 3 and S3. The detailed description of characteristic bands is provided in the Supporting Information file.

Analyzing the composites obtained after the MORPH sorption process, MORPH@MOF, we can observe certain relationships. For the composite MORPH@UiO-66 and MORPH@NU-1000, the presence of bands derived from MORPH is most evident, and the bands are characterized by considerable intensity. For example, in the MORPH@UiO-66 sample, we can observe bands at the following wavelengths: 1507 and 3055 cm^–1^. For the MORPH@UiO-67 composite, only two bands are present, 1018 and 1507 cm^–1^, confirming the presence of the drug.

There are several characteristic bands observed on the DRIFT spectrum of the NAL. The band at a wavenumber of 1392 cm^–1^ is associated with the vibration originating from the C-O-C group,^59^ while the vibration of the benzene ring is responsible for the one at 1466 cm^–1^.^60^ The obtained DRIFT spectra for NAL@MOFs confirm the effective loading of the antidote molecule into the MOF structure. Particularly significant are the two bands: at 1604 and 1725 cm^–1^, which are observed in all composites. In addition, the band at 2932 cm^–1^, originating from NAL, is visible on the spectra of NAL@UiO-66 and NAL@NU-1000.

### 3.2 MORPH Sorption Process

The efficiency of zirconium-based metal–organic frameworks used in this study was tested in the MORPH sorption process (Figures 4A, and S4A, S5A,B). This compound, classified as a potent opiate, is currently the most popular treatment for acute and chronic pain, whether in hospitals, hospices, or individual home therapy.^61^ Because of its high addictive potential and relatively easy availability, the risk of addiction, as well as intentional and unintentional overdose, is increasing.^62^ The MORPH adsorption experiments were conducted in MORPH aqueous solution and for MORPH solution in a medium simulating human body fluid (Simulated Body Fluid, SBF).

**Figure 4:**
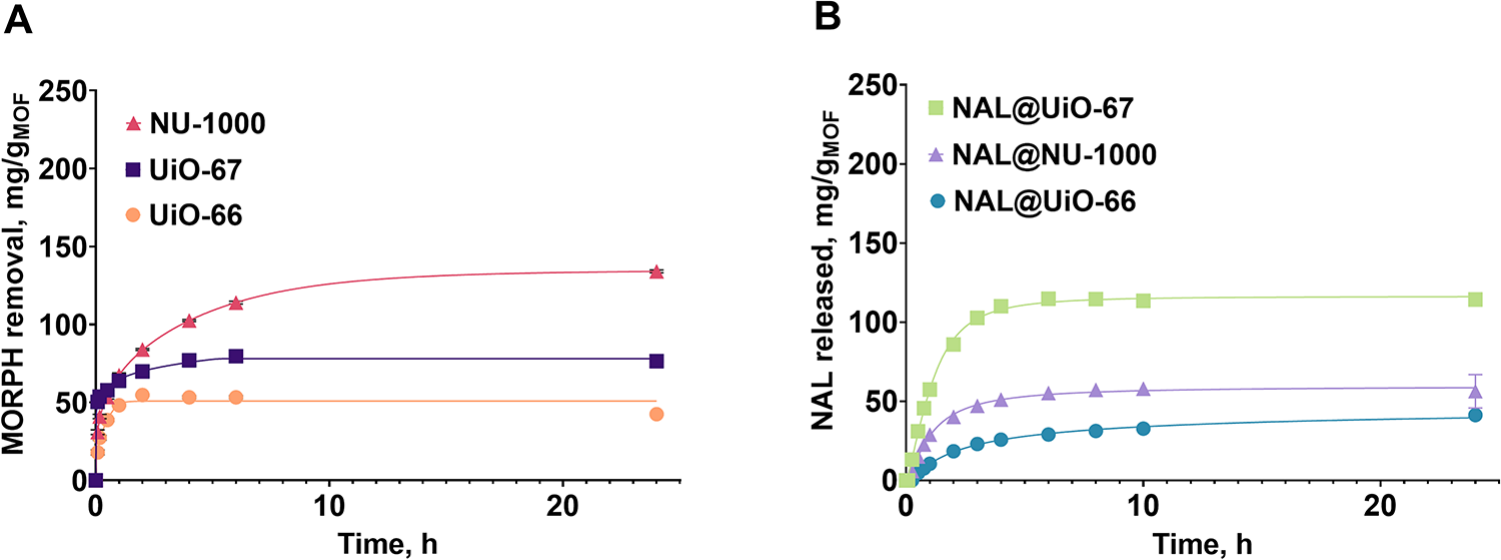
(A) MORPH sorption and (B) NAL release by MOFs in SBF solution.

Among the MOF materials used: UiO-66, UiO-67, and NU-1000, the highest efficiency calculated per gram of MOF was observed for UiO-67 and NU-1000. Their superior specific surface areas allowed them to adsorb 97 mg and 93 mg of MORPH in H_2_O per 1 g of UiO-67 and NU-1000, respectively. The UiO-66 had almost three times worse sorption, which was 32 mg of MORPH per gram of material. This is related to the percentage conversion of MORPH removal efficiency. By expression of the sorption capacity as the percentage of MORPH removals, the following results were achieved: 64 wt % for NU-1000, 21 wt % for UiO-66, and 47 wt % for UiO-67 (Figure S5A).

Regarding adsorption kinetics, it varies depending on the MOF used. For UiO-66, MORPH sorption of half the maximum amount adsorbed by the material was reached after ca. 1 h. For UiO-67, such a process was very fast, less than 5 min, while for NU-1000, more than 10 min. This emphasizes the highest efficiency of NU-1000 and UiO-67 in the mentioned process. The relationships are shown in Figure S4A.

Referring to the therapeutic doses administered, which are between 5 mg and 20 mg for chronic pain,^63^ the MOFs used can adsorb such amounts up to 30 min after application in the case of UiO-66, up to 5 min in the case of UiO-67, and NU-1000. Assuming the application of a life-threatening dose of 60 mg or more by the patient,^64^ we can adsorb all the MORPH ingested using a gram of UiO-67 and NU-1000. Although for NU-1000, complete adsorption would be achieved after a time of ca. 2 h, a significant portion of the drug would be adsorbed much faster, reducing the death risk to the patient. More than 60 mg of MORPH is adsorbed within 5 min in the case of UiO-67. Even though the efficiency of the process is high, such kinetics can be dangerous for humans. During an overdose, it is important to gradually remove the drug from the body so as not to cause a feeling of psychological shock. Such a rapid adsorption, while efficient, could be extremely dangerous. Therefore, the NU-1000 usage as a medical adsorbent seems to be a better solution. It combines both desirable characteristics: speed and controlled nature. A similar experiment was conducted for the second medium, MORPH in SBF solution (Figure 4A).

In this case, an increase in the amount of drug adsorbed was observed for each material. Also, the maximum sorption was achieved after the application of NU-1000 and was 133 mg g_MOF_^–1^, which is 90 wt %. Subsequently, for UiO-66 and UiO-67, the sorption values were about 42 mg g_MOF_^–1^ and 79 mg g_MOF_^–1^, which corresponds to 28 and 52 wt % sorption. When UiO-66 is used, half of the maximum adsorbed amount is adsorbed after 5 min, as well as a dose corresponding to the therapeutic one. For UiO-67 and NU-1000, a time of less than 5 min is sufficient. The sorption kinetics in SBF in percentages are shown in Figure S5B.

Hence, as we can see, the SBF solution supports the sorption process and gives MOFs improved sorption capacity. This may be due to the presence of ions in the solution, which contribute to a hydroxyapatite layer formation on the surface of MOFs.^57,58^ As a result, the metal–organic framework functionalized with the established hydroxyapatite more easily adsorbs compounds. This is confirmed by numerous studies on the adsorption capacity of pure hydroxyapatites.^38,57,58^ This is because they can adsorb either heavy metal ions, radioactive contaminants, or organic compounds on their surface. The presence of hydroxyapatite can promote the processes that accompany adsorption, such as ion exchange, surface complexation, or the formation of hydrogen bonds or electrostatic interactions in the case of physical adsorption.

Concerning practical aspects and future medical adsorption, MOF usage seems to be a rational and reasonable solution. Assuming the presence of an ionic environment, sorption from SBF solution reflects the conditions in the human body. Accordingly, UiO-67 and NU-1000 materials appear to be the most favorable, emphasizing the high effectiveness of NU-1000 in the situation of the need to adsorb large doses of MORPH in a short time. Moreover, NU-1000 retains more favorable MORPH sorption kinetics. UiO-67 material can adsorb large amounts, but its efficiency is lower, and its kinetics are less favorable. MORPH is adsorbed very quickly for the first hour of the process, and its sorption is marginal. In the case of NU-1000, we observe continuous and gradual, and, consequently, efficient sorption of the drug. MORPH sorption from the SBF solution is shown in Figure 4A.

For each MOF and MORPH sorption performed for both aqueous solution and SBF, pseudo-first-order and pseudo-second-order kinetics were calculated, as shown in Figure S7. Referring to the most effective sorption of MORPH obtained after using NU-1000, an additional experiment was performed to determine the Langmuir and the Freundlich isotherms for the mentioned process. For this purpose, different initial concentrations of MORPH of 0.1, 0.2, and 0.4 mg mL^–1^ were prepared. Additionally, the Langmuir and the Freundlich isotherms are presented in Figure S8.

### 3.3 NAL Release Study

The release of NAL was carried out from the three composite materials, NAL@UiO-66, NAL@UiO-67, and NAL@NU-1000, into the medium of distilled water and SBF solution (Figures 4B, S4 and S5).

The kinetics of NAL release into distilled water are quite similar for all composites. The highest efficiency was observed for NAL@NU-1000, from which more than 182 mg g_MOF_^–1^ of the antidote was released. For NAL@UiO-66 and NAL@UiO-67, the amounts of the released compound were lower, reaching 116 and 106 mg g_MOF_^–1^, respectively, and a rapid drug release was visible. For NAL@UiO-66 and NAL@NU-1000, we observe the release of half of the maximum amount of the compound after 30 min of testing. For NAL@UiO-67, this time is extended to 45 min. Despite this, the favorable and gradual kinetics of NAL release were preserved. These relationships are shown in Figure S4B. Based on the amounts of NAL, given not in mg of NAL per 1 g of MOF, but in wt.% (Figure S5C,D), we can see significant differences. These are due to differences in the loading of the drug into the MOF, and the actual amounts of NAL in the structure of the different MOFs are given in Table 1. In the case of the antidote release into SBF, we observe a completely different relationship. First, there is an evident increase in the release efficiency of NAL from the NAL@UiO-67 composite to about 115 mg g_MOF_^–1^. On the other hand, a significant decrease in release efficiency was observed for the other two composites. The amounts of released NAL are 47 mg g_MOF_^–1^ and 56 mg g_MOF_^–1^ for NAL@UiO-66 and NAL@NU-1000, respectively.

This relationship is related to the inhibitory effect of ions dissolved in the SBF solution. The presence of Cl^–^, SO_4_^2–^, HCO_3_^–^, and HPO_4_^2–^ ions causes the formation of additional interactions between the mentioned ions and the metal–organic framework.^65^

Consequently, this hinders the drug release from the MOF framework and reduces the efficiency of the process. A different relationship is observed if the metal–organic framework is inherently unstable in ionic solutions.^66^ This is the case with UiO-67, which exhibits low chemical resistance. Already after the introduction of NAL, a loss of the crystallinity of the structure and, thus, a loss of stability was observed, as confirmed by PXRD analysis of the NAL@UiO-67 composite (PXRD results Figure S1A). The structure of NAL@UiO-67 collapsed, so the dissolved ions did not interact with the backbone and perhaps with NAL, which contributed to a significant improvement in process efficiency.^65–67^ We have already observed a similar relationship in our previous studies. The kinetic profiles of the process of NAL release into SBF solution are shown in Figure 4B. Referring to the therapeutic amounts,^68^ we notice that in each case, regardless of the medium or composite used, the therapeutic doses are reached and even exceed the daily intake. It means that the release is efficient, and in practical application, it is possible to use smaller amounts of MOF carrier, making the solution economically attractive.

### 3.4 Simultaneous MORPH Sorption Experiment on NU-1000 and NAL Release from NAL@UiO-67

A common experiment was carried out to test the applicability of metal–organic frameworks in two aspects simultaneously, as a MORPH adsorbent and an antidote delivery system. The NAL@NU-1000 was used as a drug delivery system and MORPH adsorbent at the same time. Due to the large specific surface area and high pore volume, this material seems to be effective in both. Nevertheless, considering a low efficiency in NAL delivery from NAL@NU-1000 to SBF solution, and, on the other hand, good adsorption MORPH capacity, we proposed an alternative approach of a MOF mixture as detailed in the following. The NU-1000 was chosen as the adsorbent for MORPH, while the NAL@UiO-67 composite was to serve as the antidote delivery system due to its favorable and gradual, as well as efficient release kinetics. Additionally, the specific property of UiO-67, exhibiting structure collapse upon exposure to SBF, was an additional criterion to avoid additional readsorption of previously released NAL to the medium. The results of simultaneous sorption of MORPH and release of NAL are shown in Figures 5 and S6.

**Figure 5:**
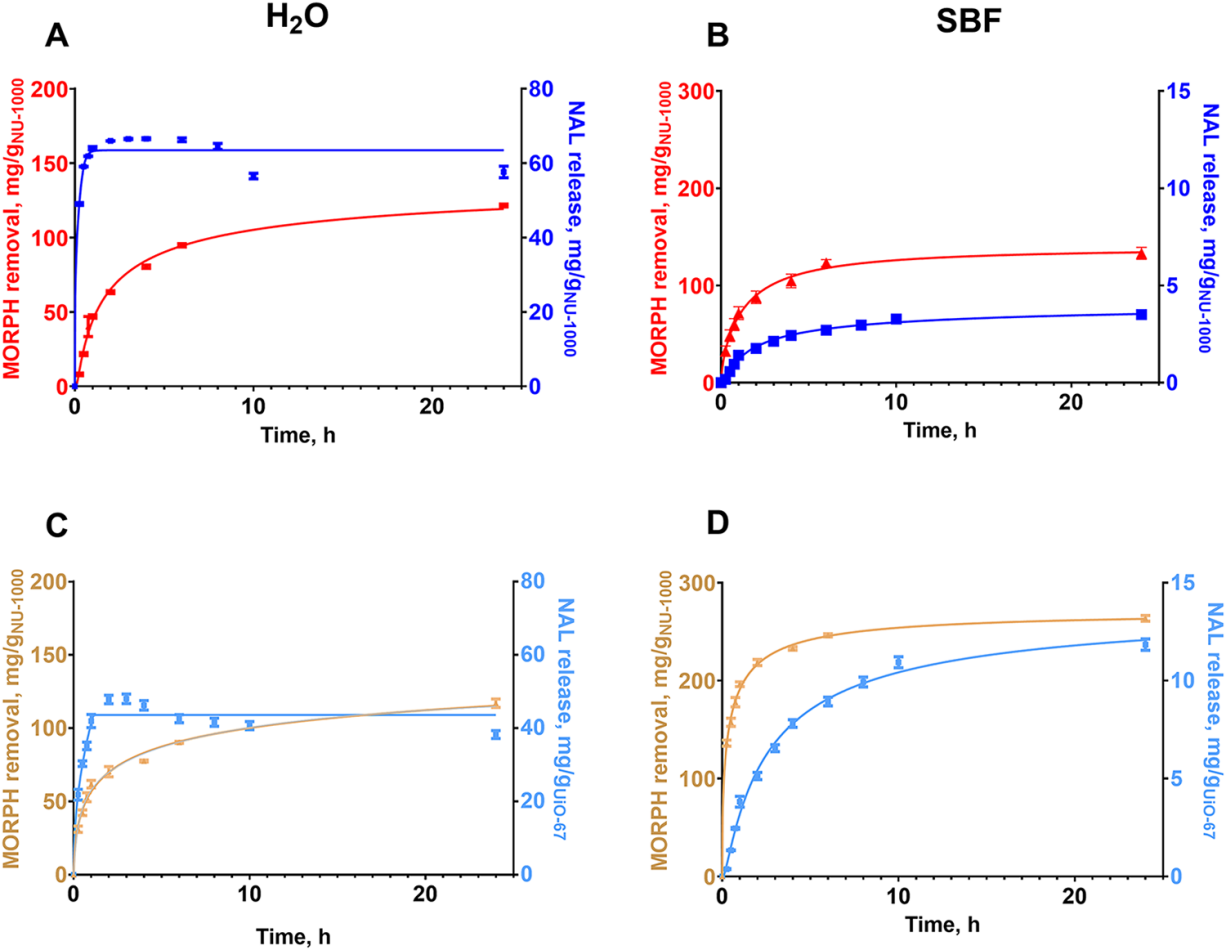
Simultaneous MORPH sorption experiment and NAL release from NU-1000 (A, B) and from mixture of NU-1000 (MORPH adsorption) and UiO-67 (NAL release) (C, D) in H_2_O (left) and SBF (right) environment.

Figure 5A,B show adsorption kinetics and release profile on NAL@NU-1000 in a simultaneous experiment on H_2_O and SBF. We observed that the level of MORPH sorption on NU-1000 is the same in both media and it is equal to ca. 100 mg g_NU‑1000_^–1^. Additionally, kinetics is gradual and prolonged, which is obligatory in medical applications. For NAL release, the efficiency of delivery is 60 mg g_NU‑1000_^–1^ in H_2_O and only ca. 4 mg g_NU‑1000_^–1^ in SBF. A significant decrease in the amount of drug release is associated with the inhibiting effects of dissolved ions and the interaction between ions and MOF structures. The limited dosages of NAL release may be due to the high stability of NAL@NU-1000 composite or even subsequent adsorption of NAL on the material surface. Therefore, the above-mentioned second experiment with NU-1000 and NAL@UiO-67 was conducted and is described below.

When the experiment was conducted in distilled water, we observed 50% of MORPH adsorption and 25% of NAL release. In the case of NAL release, most of the drug is released after about 45 min. It may be observed that in the case of NAL, its release was fast and reached its maximum in the first 2 h of the experiment, and gradually decreased and reached a plateau after 15 h of the experiment. MORPH sorption, on the other hand, was gradual, and after about 2 h, half of the maximum amount was adsorbed. An analogous experiment performed in SBF solution as a medium is characterized by completely different results. Although there is a significant decrease in the efficiency of NAL release, up to 8%, the process occurs gradually and in a controlled manner. We observe slow release of the drug and prolongation of the process up to 24 h. During this time, we observe the steady release of NAL. This relationship is very favorable for developed materials, considering the short half-life of NAL, related to its short half-life. In addition, NAL administration is characterized by the need for continuous repeat dosing to avoid the recurrence of overdose effects. Here, through prolonged release, the need for repeated dosing is eliminated. The amounts released are about 12 mg per 1 g of composite after 24 h. This was found to be enough for potential overdose treatment applications.

Based on the release of NAL, doses of 0.1–0.3 mg per kg of body weight are usually administered every 1–2 h approximately. From NAL@UiO-67, 12 mg is released over 24 h, and the release is gradual, ensuring that the therapeutic dose is maintained in the patient’s plasma. The suggested carrier appears very efficient in real-world applications, considering the therapeutic effect and the release kinetics. In the case of MORPH sorption, the process efficiency in SBF is higher than 88%. It is significantly higher than for sorption in water, which is most likely related to the formation of a hydroxyapatite layer on the MOF surface.^58,69,70^ This relationship was observed in the sorption study and described above. A material of 1 g can adsorb about 270 mg of MORPH. It is significantly more than the lethal dose for humans. Therefore, the NU-1000 material can adsorb a dangerous human dose, which reliably protects against overdose. In addition, the adsorption kinetics are favorable and gradual. It eliminates the effect of physiological shock for humans associated with too-fast removal of the drug. Here, gradual but very effective removal occurs throughout the process, 24 h, although it is most effective during the first 10 h.

In summary, the proposed solution, i.e., the use of NU-1000 as a medical adsorbent and NAL@UiO-67 composite as an antidote delivery system, is an effective way to combat opioid overdose. The use of the aforementioned materials has a dual effect of simultaneously adsorbing up to 270 mg of MORPH and releasing ca. 12 mg of NAL. These amounts are adequate to reverse the result of an overdose. The proposed dual detoxification system is more than safe, involving a controlled, gradual, and slow process of MORPH sorption and NAL release. The solution is thus both commercially viable and future-proof. The lack of medical adsorbents for the removal of drugs such as opioids, including MORPH, over and above the problems of NAL delivery, underscores the great role and innovative nature of the proposed solution. Using the presented system, we can not only effectively and safely remove MORPH from the body but also ensure a gradual release of NAL with a single administration of the drug. Thus, we eliminate the need to repeat the dose every 1–2 h, approximately. Hence, we contribute to reducing the mortality rate of people due to overdose, which is a common occurrence during the current “opioid epidemic”.

### 3.5 DFT Modeling

For NU-1000, the sorption of MORPH was stronger in wide channels, while for NAL hydrochloride (NAL), the narrow channels resulted in stronger adsorption. The effect of the sorption of two organic molecules in the same large channel of NU-1000 gives a sorption stronger by 0.120 eV with respect to the sorption of MORPH hydrochloride or NAL independently (Table 2). It should be emphasized here that, due to the nature of the basis set used in the simulations (plane waves), the basis set superposition effect (BSSE) is absent.

Introduction of the polar solvent destabilizes the adsorbate molecules, and for the system of NAL + MORPH in NU-1000, the effect of the sorption of two organic molecules in the same large channel of NU-1000 is a sorption weakening by 0.193 eV, see Table 3, which also comprises the loading expressed in % wt.

**Table 3:** Energetics of MORPH and NAL Sorption

adsorbate molecule	*E*_ads_ (vacuum)/eV	*E*_ads_ (water)^a^/eV	*n*_molecules_ ^b^ (calculated)	*n*_molecules_ ^c^ (exp.)
NU-1000				
MORPH, narrow channel	–1.435	–1.180	24 (8)	3.54
MORPH, wide channel	–1.695	–1.091		
NAL, narrow channel	–1.911	–1.298	20 (6.67)	8.55
NAL, wide channel	–1.485	–0.855		
NAL + MORPH, wide channel	–3.300	–1.753	16 (5.33)	
UiO-67				
MORPH, str. 1	–1.687	–1.101	3 (3)	1.08
MORPH, str. 2	–0.996	–0.727		
MORPH str. 3	–1.392	–1.000		
NAL, str. 1	–1.121	–0.872	2 (2)	1.02
NAL, str. 2	–0.964	–0.651		

a Calculations performed with polarizable continuum with ε = 80.

b Calculated within the rigid host approximation (in parentheses: per a single Zr_6_ cluster).

c Values calculated from the experimental % wt (Table 1).

The sample DFT-optimized structures of MORPH molecules sorbed in NU-1000 large and small channels are presented in Figure 6A,C, respectively. The fragmentary views of the closest neighborhood of the adsorbed molecules are shown in inserts (Figure 6B,D). The other structures of MORPH@MOF and NAL@MOF are presented in the Supporting Information file (Figure S9).

**Figure 6:**
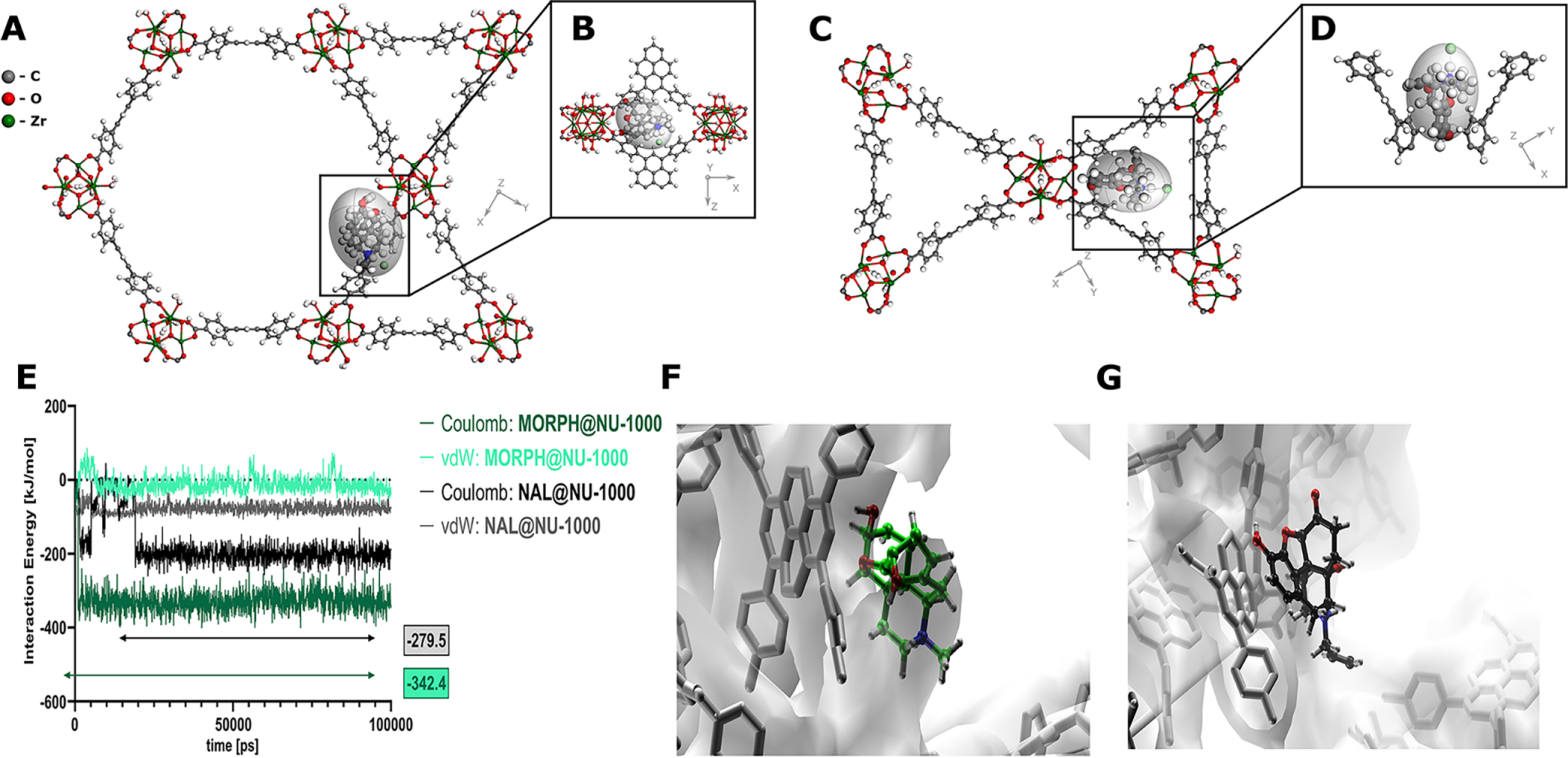
DFT-optimized structures of MORPH@NU-1000 adsorbed in the large (A, and magnified fragment, B) and small channels (C, and magnified fragment, D). Results of molecular dynamics simulation on the spontaneous aggregation of MORPH and NAL within the pores of NU-1000 (E), changes of interaction energies between the NU-1000 and MORPH or NAL (NAL), and close-up views of the complexes with MORPH (F) or NAL (G) molecules in the channel of MOF; the structures are representative after hierarchical clustering based on RMSD; water molecules and hydrogen atoms of MOF are not shown for clarity.

### 3.6 Molecular Dynamics

To better understand how the NAL@NU-1000 complex can support the treatment of MORPH overdose, a series of 100 ns molecular dynamics (MD) simulations were performed (Figure 6E–G, Figures S10–S12, Movies S1, S2 and S3). The initial configurations were constructed by randomly placing the MORPH, NAL, or both molecules within the DFT-preoptimized NU-1000 and filling the TIP3P water molecules.

In general, both drugs are absorbed within the pores of NU-1000. However, MORPH exhibits greater conformational flexibility, as reflected in the calculated mean squared displacement (MSD) values and diffusion coefficients (see the Supporting Information, Figure S10). Energy analysis of the MD trajectories when both molecules were adsorbed in the MOF structure (Figure 6E) indicates that MORPH is more strongly bound. The nature of this interaction is primarily polar and results from noncovalent (dispersion) interactions between the hydroxyl groups of MORPH and the pyrene system, specifically π-polar interactions associated with the quadrupole moment of the π system of pyrene. NAL exhibits weaker binding in NU-1000, and visual inspection of the formed complex suggests that, in this case, π-π stacking interactions with the aromatic system of 1,3,6,8-tetrakis­[*p*-benzoic acid]­pyrene play a dominant role. Interestingly, when the simulations were conducted separately for each drug, NAL appeared to form a more stable complex with NU-1000 (SI, Figures S8 and S9). The nature of these interactions is similar to those described above, with one key difference: in the case of MORPH, due to the presence of two hydroxyl groups, two slightly different binding variants were observed, with minimal energy differences between them.

These findings suggest a competitive binding mechanism where both molecules compete for the binding site. However, unlike their interaction with the opioid receptor, the roles are reversed—MORPH appears to bind more readily to NU-1000 in the presence of NAL. In light of the above considerations, the solution proposed in this study seems to be both rational and well-founded. Specifically, implementing a dual adsorption–desorption system incorporating two distinct materials offers a promising approach. Within this system, NU-1000 is utilized for the adsorption of MORPH while simultaneously facilitating the desorption of the detoxifying agent from the physiologically unstable UiO-67. This dual functionality not only addresses the primary challenge but also enhances the overall efficiency and stability of the process. This study provides new insights into the detoxification process following opioid overdoses.

### 3.7 In Vitro

The experiment was designed to model MORPH dependence at the cellular level by exposing cells to progressively increasing doses of MORPH for eight consecutive days (10, 15, 20, 25, 30, 35, 40, and 50 mg L^–1^) (Figure 7). Before each change in concentration, cells were passaged to maintain optimal growth conditions and reduce variability. This stepwise dose escalation aimed to mimic the gradual development of tolerance and dependence observed in vivo.

**Figure 7:**
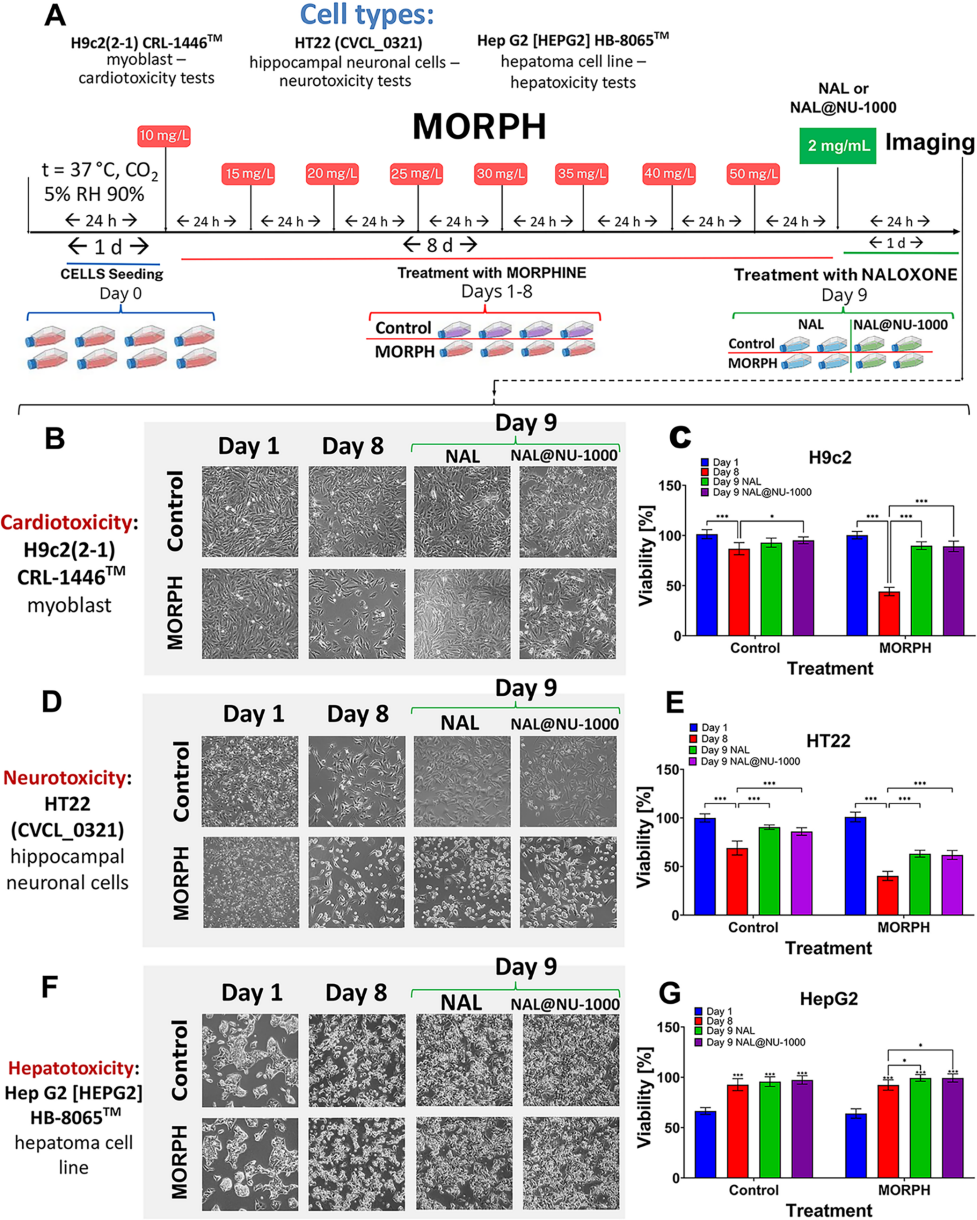
Experimental scheme and evaluation of MORPH dependence and then NAL and NAL@NU-1000 effects on cardiotoxicity, neurotoxicity, and hepatotoxicity. (A) Experimental timeline. (B–G) Representative bright-field images and quantitative viability analysis for: (B, C) H9c2 myoblasts (cardiotoxicity assay), (D, E) HT22 neurons (neurotoxicity assay), and (F, G) HepG2 hepatocytes (hepatotoxicity assay). Data are presented as mean ± SD (*n* = 6). Statistical significance was determined by two-way ANOVA, Tukey post hoc: *p < 0.05, **p < 0.01, ***p < 0.001.

After eight days of MORPH exposure, a detoxification phase was initiated using either free NAL, a commonly used opioid antagonist, or NAL encapsulated within the NU-1000 metal–organic framework (NAL@NU-1000). One of the limitations of standard NAL therapy is its short duration of action, which can lead to renarcotization after initial reversal. Controlled release of NAL from the MOF carrier may help overcome this drawback by sustaining therapeutic levels over a longer period. Moreover, NU-1000 possesses an exceptionally high specific surface area, making it highly suitable for drug loading and prolonged delivery applications.

The toxic effects of MORPH were most prominently observed in neuronal (HT22, *p* < 0.001) and cardiac (H9c2, *p* < 0.001) cells. In contrast, hepatic cells (HepG2) did not exhibit reduced proliferation upon MORPH exposure and, rather, on the contrary, these cells continued to grow without apparent disturbance, suggesting a higher resistance to MORPH-induced cytotoxicity. Surprisingly, in hepatic cells, both NAL and NAL@NU-1000 significantly increased cell viability, but only in the MORPH-dependent group (*p* < 0.05). Among all tested cell lines, HT22 cells proved to be the most sensitive to MORPH. Additionally, a noticeable decline in proliferation was also observed in the HT22 control group after 8 days (*p* < 0.001), likely due to the daily passaging process, which may have negatively impacted their growth. However, both free NAL and NAL@NU-1000 significantly improved cell viability in all groups, with particularly notable recovery in MORPH-dependent cells (*p* < 0.001). In cardiac cells, a similar trend was observed in the MORPH-treated group (*p* < 0.001), although the control group did not exhibit such a marked decrease in viability after 8 days (*p* < 0.05). These results highlight the high sensitivity of cardiac cells to MORPH toxicity. Importantly, in these cells, NAL@NU-1000 appeared to be slightly more effective than free NAL in restoring viability (*p* < 0.05), suggesting potential benefits of the sustained-release formulation.

### 3.8 In Vivo Studies

#### 3.8.1 Zebrafish

To determine the influence of MOFs on MORPH-induced toxicity in zebrafish larvae, locomotor activity and heart rate parameters were evaluated. The results are shown in Figure 8.

**Figure 8:**
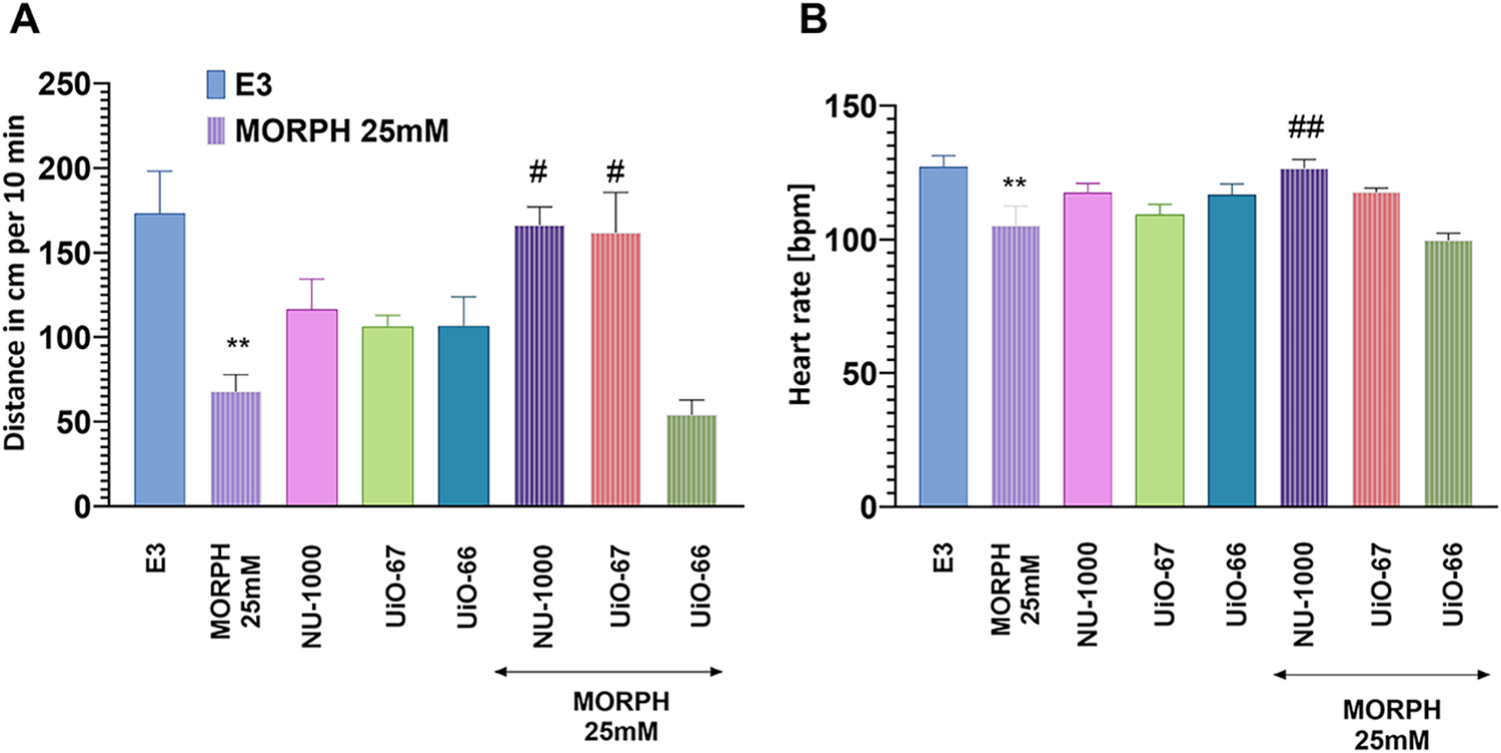
Influence of MORPH (25 mM) and selected metal–organic frameworks (MOFs: NU-1000 (2.5 mg mL^–1^), UiO-66 (4 mg mL^–1^), UiO-67­(4 mg mL^–1^)) on zebrafish larvae assessed by measuring two parameters: (A) average distance (in cm) moved during the 10-min light phase, and (B) heart rate (beats per minute), recorded over 1 min. Data expressed as mean ± SEM (*n* = 10–12). Statistical significance evaluated using Tukey’s post hoc test: **p < 0.01, vs E3 control; ^#^ *p* < 0.05, ^##^ *p* < 0.01, vs MORPH-treated group.

In the locomotor activity test, a two-way ANOVA revealed a significant effect of MORPH treatment (*F*(3, 85) = 4.529, *p* = 0.0054) and a significant interaction effect (*F*(3, 85) = 9.323, *p* < 0.0001), while no effect was observed for MOFs alone (*F*(1, 85) = 0.8278, *p* = 0.3655). Bonferroni’s post hoc test confirmed that MORPH at 25 mM significantly reduced the total distance moved by the larvae (*p* < 0.01). Importantly, coincubation of MORPH with the tested MOFs (NU-1000 or UiO-67) significantly reversed the MORPH-induced reduction in locomotor activity (*p* < 0.05 vs MORPH group).

A two-way ANOVA revealed significant effects of MORPH (*F*(3, 70) = 4.916, *p* = 0.0037), interactions between both factors (*F*(3, 70) = 8.128, *p* = 0.0001) without effect of MOF treatment (*F*(1, 70) = 2.680, *p* = 0.1061) on heart rate. Bonferroni’s post hoc test confirmed that MORPH at a concentration of 25 mM significantly reduced heart rate in zebrafish larvae (*p* < 0.01). Co-treatment with NU-1000 effectively prevented the MORPH-induced reduction in heart rate (*p* < 0.01).

#### 3.8.2 Rodents

In the study of rodents, we focused on the potential modulation of withdrawal symptoms and locomotor responses following acute and chronic MORPH administration to investigate the effects of selected MOFs on MORPH-induced hyperlocomotion and physical dependence. Figure 9 indicates the effects of acute administration of MORPH and MOFs on locomotor activity (two-way ANOVA: MORPH pretreatment [*F*(1, 66) = 7.364; *p* = 0.0085], MOFs treatment [*F*(3, 66) = 2.117; *p* = 0.1065] and interactions effect [*F*(3, 66) = 4.616; *p* = 0.0054]). The posthoc Bonferroni’s test showed that MORPH at the dose of 0.025 mg kg^–1^ (*p* < 0.001) and UiO-66 (*p* < 0.05) significantly increased locomotor activity. Co-administration of MORPH with NU-1000 and UiO-67 statistically significantly decreased locomotor activity when compared with the MORPH-treated group (*p* < 0.01).

**Figure 9:**
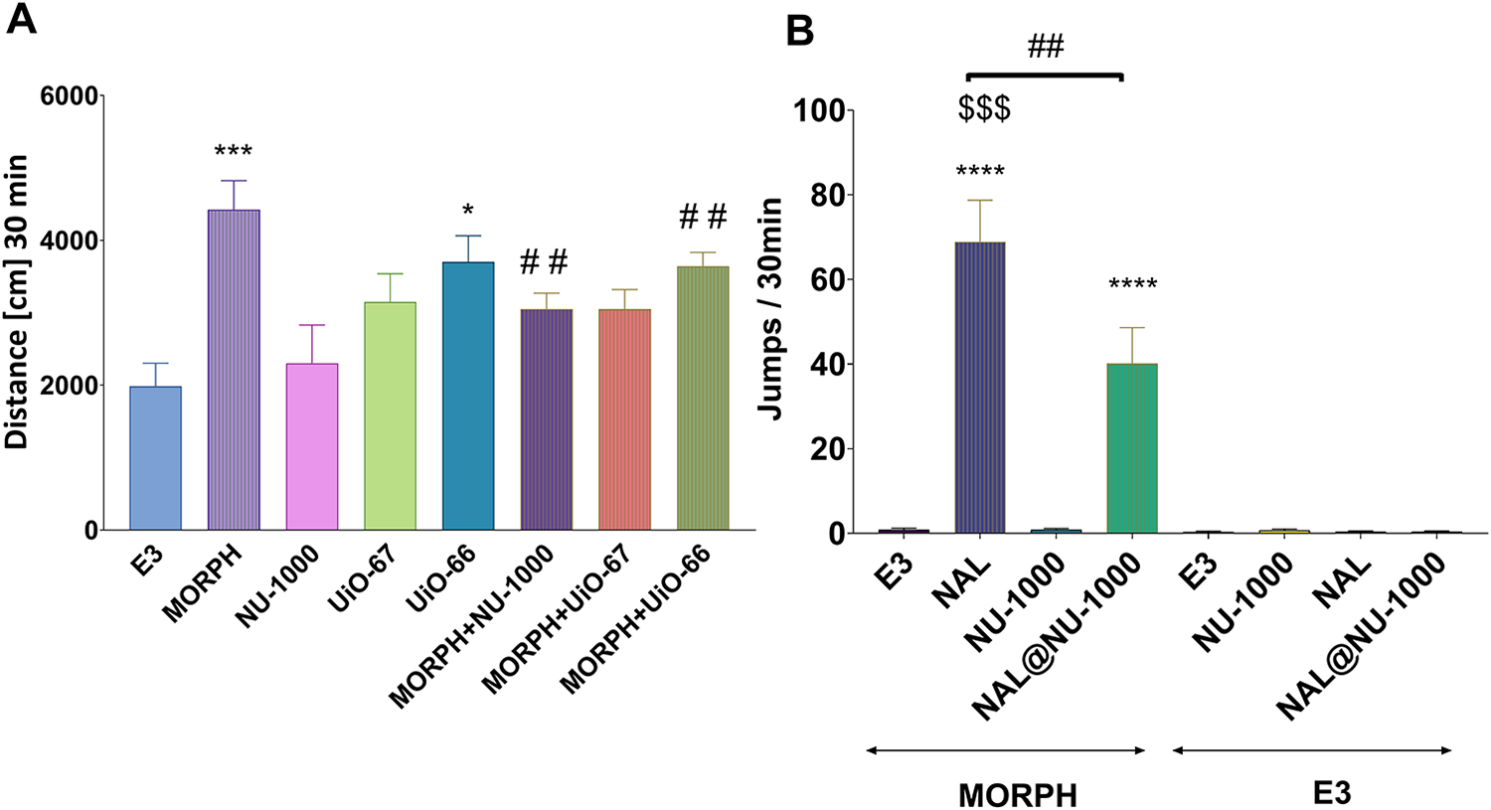
(A) Effect of MORPH (MORPH 0.025 mg kg^–1^, i.v.) and metal–organic frameworks (MOFs: NU-1000: 0.04 mg kg^–1^, UiO-67: 1.55 mg kg^–1^, and UiO-66: 1.85 mg kg^–1^, i.v.) administered separately or in combination on spontaneous locomotor activity in mice. Mice were injected with MORPH and, 15 min later, with MOFs. Locomotor activity (number of interruptions of light beams) was recorded for 30 min. Data is presented as the means ± SEM, *n* = 9–10, ***p < 0.001, *p < 0.05 vs saline-treated group; ^##^ *p* < 0.01 vs MORPH-treated group (post hoc Bonferroni’s test), (B) the effect of NAL@NU-1000 (mg/kg, i.p.) on MORPH (MORPH) dependence, in mice. MOPRH was administered for eight consecutive days (10, 15, 20, 25, 30, 35, 40, and 50 mg kg^–1^), twice a day. On the ninth day, MOPRH (50.0 mg kg^–1^) was administered first, and 1 h later, NAL (2 mg kg^–1^, i.p.) was injected. The animals were immediately placed in glass cylinders, and the number of MOPRH jumping behaviors was recorded for 30 min. To study the effect of NAL@NU-1000 on the expression of MOPRH withdrawal signs, NAL@NU-1000 was administered on day ninth, 30 min after MOPRH injection, and the number of MOPRH jumping behaviors was observed after another 30 min for half an hour. The results are shown as the average number of jumping behaviors ± SEM ^$$$^ *p* < 0.001 vs vehicle group, ****p < 0.0001 vs MOPRH group, ^##^ *p* < 0.01 vs MOPRH + NAL group, (Bonferroni’s test).

#### 3.8.3 Physical Dependence and Effects of MOFs on the Expression of MORPH Withdrawal Signs in Mice

Two-way ANOVA revealed significant differences in the studied mice (MORPH effect: *F*(3, 50) = 66.05, *p* < 0.0001; NU-1000 effects: *F*(1, 50) = 4.443, *p* = 0.0401; interaction: *F*(3, 50) = 4.72, *p* = 0.0056). The administration of NAL in mice chronically treated with MORPH induced a substantial increase (*p* < 0.0001) in the number of jumping behaviors compared to MORPH-treated mice. In contrast, the administration of NAL@NU-1000 (20.04 mg kg^–1^, i.p.) significantly reduced (*p* < 0.01) the number of jumping behaviors compared to the MORPH group. NAL@NU-1000 alone did not produce any jumping behavior in mice treated with either saline or saline + NAL. Results for NAL@UiO-66 and NAL@UiO-67 are presented in the Supporting Information file (Table S2).

### 3.9 LC-MS/MS Measurements and Quantification of MORPH in Brain and Serum

To comprehensively understand the influence of MOF on MORPH pharmacokinetics, both central and peripheral drug distributions must be considered (Figure 10).

**Figure 10:**
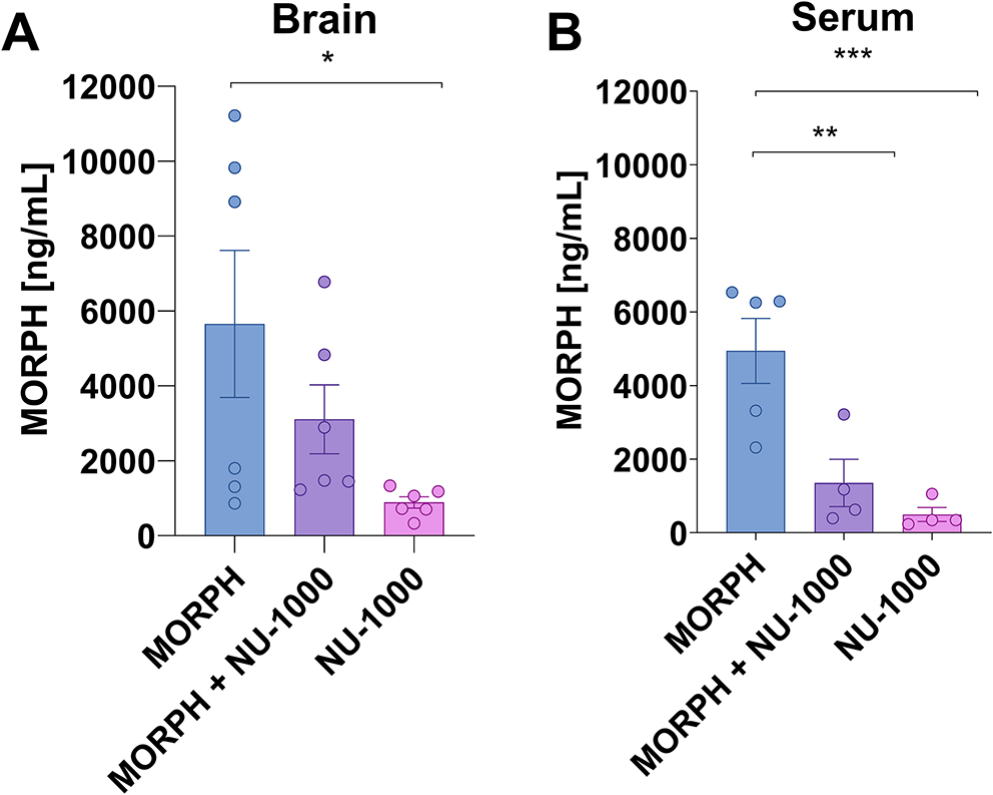
Effect of NU-1000 (0.04 mg/kg, i.v.) on MORPH (0.025 mg kg^–1^, i.v.) levels in the brain (A) and serum (B) in mice acutely injected with MORPH. MORPH was injected first, followed by MOF administration 15 min later. Mice were decapitated, and blood and brain samples were collected one hour after MORPH injection. Data are expressed as mean ± SEM (*n* = 4–6). *p < 0.05 between NU-1000 and MORPH-treated group (Student’s *t*-test).

Unpaired *t*-test revealed a trend toward significant differences in MORPH concentration in the brain (*F*(5, 5) = 2.3681, *p* = 0.2674). A statistically significant effect was observed in serum levels (*F*(4, 3) = 2.3681, *p* = 0.0168). Co-administration of NU-1000 with MORPH significantly reduced the serum concentration of the opioid in mice (*p* < 0.05). Although a decrease in MORPH levels was also observed in the brain, this difference did not reach statistical significance (*p* > 0.05).

MORPH’s effect on the central nervous system extends beyond analgesia; it also significantly influences locomotor activity. In zebrafish larvae, MORPH acts predominantly on the immature central nervous system, leading to sedation and hypoactivity via enhanced μ-opioid receptor signaling and suppression of neuromuscular circuits. Conversely, in rodents, low-to-moderate doses of MORPH often induce locomotor activation by stimulating reward-related dopaminergic pathways, followed by sedative effects at higher doses. This species- and developmental-dependent divergence likely reflects differences in the maturation of neural circuits, opioid receptor distribution, and pharmacokinetic profiles. Our study confirmed MORPH-induced hypolocomotion in 5 dpf zebrafish larvae, whereas acute MORPH administration at the dose of 0.025 mg kg^–1^ significantly increases locomotor activity in mice. In rodents, this hyperlocomotion serves as a behavioral marker of the drug’s rewarding properties, which contribute to its addictive potential. Thus, our study investigated whether specific MOFs could modulate MORPH’s effects on locomotor activity. Among the MOFs tested, NU-1000 and UiO-67 showed the most promising results. When coadministered with MORPH, both MOFs significantly reduced the drug-induced locomotor changes in both species.

Furthermore, MORPH, consistent with its known depressant effects on the central nervous system, significantly reduced cardiac activity in zebrafish. Of particular note is the observation that cotreatment with NU-1000 mitigated the MORPH-induced reduction in heart rate. This suggests a possible protective or modulating role of NU-1000 in opioid-related cardiac effects. MORPH, a widely used opioid analgesic, is essential for pain management but is associated with significant risks, including the development of physical dependence and addiction. A critical challenge in treating MORPH addiction is managing the withdrawal symptoms. Traditional approaches to mitigating these symptoms are limited and can lead to further complications. Interestingly, NAL@NU-1000 administration in MORPH-dependent mice not only attenuated the number of jumps, a key withdrawal symptom, but also did not induce any withdrawal-like behaviors when administered alone in vehicle-treated mice. It indicates a potential specificity in NU-1000s action, where it mitigates the withdrawal symptoms in the context of MORPH dependence without affecting baseline locomotor activity in nondependent animals.

Our results also demonstrate that coadministration of NU-1000 significantly alters the pharmacokinetics of MORPH in vivo (Figure 10A,B). Specifically, a single dose of NU-1000 led to a statistically significant reduction in MORPH concentration in the serum (Figure 10B). This suggests that NU-1000 may facilitate systemic clearance of MORPH. Although a decreasing trend in MORPH concentration was also observed in brain tissue following NU-1000 treatment, the difference did not reach statistical significance (Figure 10A). Nevertheless, the observed serum reduction indicates that NU-1000 may alter the biodistribution profile of MORPH and possibly reduce its central nervous system exposure over time. The ability of NU-1000 to selectively target the pathological states induced by MORPH dependence makes it a promising candidate for further exploration as a supportive therapy in opioid addiction treatment.

### 3.10 In Vivo Biodistribution and Accumulation of MOFs

Given the observed alterations in MORPH pharmacokinetics following NU-1000 administration, it seems possible that these effects are partially the consequence of the biodistribution profile of the MOFs themselves. To study this relationship, in vivo fluorescence imaging and ex vivo organ quantification were performed to assess the systemic fate of the three MOFs used in this study (Figure 11).

**Figure 11:**
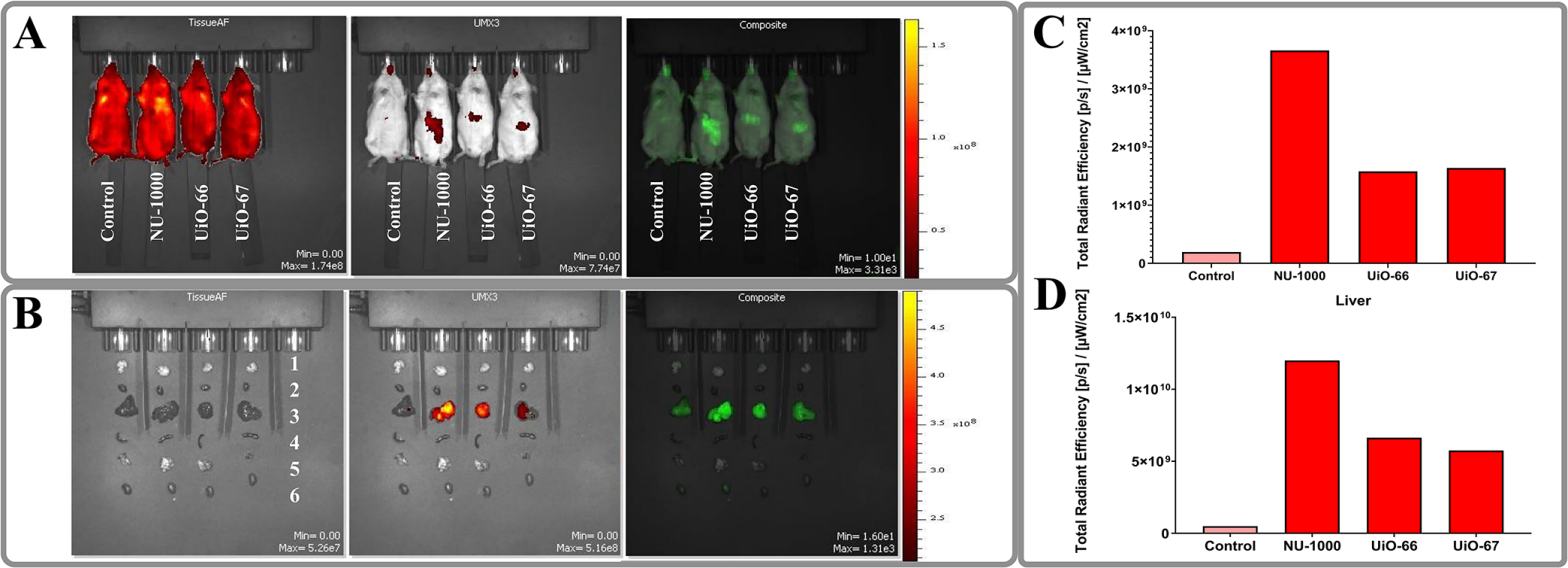
In vivo fluorescent imaging allows monitoring the MOF distribution during the MORPH removal via MOFs; (A) 24 h post injection image; (B) *postmortem* tissue image 24 h post injection; 1—brain, 2—heart, 3—liver, 4—spleen, 5—intestine, 6—kidney; (C) Quantification of in vivo fluorescence as Total Radiant Efficiency [p/s]/[μW cm^–2^] following administration of fluorescently labeled metal–organic frameworks (MOFs) in mice; (D) quantification of organ (liver) fluorescence as Total Radiant Efficiency [p/s]/[μW cm^–2^] following administration of fluorescently labeled metal–organic frameworks (MOFs) in mice.

Fluorescence imaging at 24 h postinjection (Figure 11A) demonstrates distinct biodistribution of the three MOFs studied (e.g., NU-1000, UiO-66, UiO-67). All treated animals show clear systemic fluorescence signals compared to controls, indicating that the MOFs circulated and distributed through the bloodstream. Quantitative analysis (Figure 11B) of total radiant efficiency shows that NU-1000 yields the highest fluorescence, probably through prolonged circulation time, slower clearance, or enhanced uptake by organs. Ex vivo organ imaging (Figure 11C) reveals dominant accumulation in the liver, with additional, weaker signals in the spleen and kidneys, in line with classical nanoparticle clearance routes. Quantification of hepatic fluorescence (Figure 11D) confirms the trend: NU-1000 > UiO-66 > UiO-67 in intensity, suggesting differential hepatic sequestration behavior among the MOFs. In summary, while all MOFs in this study reach systemic circulation, their differences in fluorescence intensity in the liver indicate that their structural or surface differences strongly modulate hepatic uptake and clearance. Among them, NU-1000 demonstrated the best balance between stability, systemic persistence, and organ targeting, highlighting its potential as a versatile and biocompatible nanoplatform for drug delivery. Further optimization could minimize off-target accumulation and enhance pharmacological precision, opening the way to preclinical translation and possible clinical evaluation of MOF-based therapeutic systems.

## 4 Conclusions

In this work, a dual detoxification system based on zirconium metal–organic frameworks (Zr-MOFs) acting as an efficient morphine adsorbent (NU-1000) and a naloxone cargo (UiO-67) for the prevention and treatment of opioid overdose was proposed.

Three model representatives of Zr-MOFs, including UiO-66, UiO-67, and NU-1000, were synthesized and comprehensively characterized in terms of their physicochemical properties, molecular nature, adsorption/release kinetics, toxicity, and metabolic pathways in living organisms. Among tested MOFs, NU-1000 exhibited the highest sorption performance toward morphine (MORPH), with capacities of 93 mg g^–1^ in water and 133 mg g^–1^ in simulated body fluid (SBF), corresponding to 64 and 90% removal efficiencies, respectively. Considering the lethal dose of morphine, the obtained adsorption capacities achieved by NU-1000 confirm their possible implementation as a MORPH adsorbent in emergency overdose cases. In contrast, lower MORPH adsorption capacities equal to 21 and 47% in water, and to 28% and 52% in SBF environment, together with characterization results, clearly indicate the role of pore size and topology in efficient drug removal. Similarly, naloxone (NAL) release from NAL@UiO-66 showed the most efficient delivery in water, while NAL@UiO-67 exhibited a slower, sustained release (up to 76%) in SBF solution. A combined adsorption-release experiment verified the possibility of the dual detoxification concept presented in this study, where a physical matrix of selective NU-1000 acting as MORPH adsorbent and UiO-67 as an efficient NAL cargo was proposed. In a simultaneous MORPH adsorption and NAL release experiment, 88% MORPH removal and 8% NAL release were achieved, confirming the synergistic function of both MOFs. It must be emphasized that, despite the partial degradation of UiO-67 structure, it showed prolonged NAL delivery, minimizing the possibility of NAL readsorption in the partially degraded structure. It was also proved that the enhanced adsorption of MORPH from SBF solution originated from hydroxyapatite that was formed on the MOF surface.

The experimental results were supported by DFT modeling, revealing that for NU-1000, the sorption of MORPH was stronger, and the accumulated B.O. (descriptor of the bond strength) between the adsorbate and the adsorbent was higher in wide channels, while for NAL hydrochloride (NAL), the narrow channels resulted in stronger adsorption and higher B.O. The effect of the sorption of MORPH + NAL in the wide channel of NU-1000 gives a sorption stronger by 0.120 eV with respect to the sorption of MORPH or NAL independently. The analogous synergy holds for B.O. The introduction of the polar solvent reverses this trend and destabilizes the adsorbate molecules. The sorption of NAL + MORPH in the wide channel of NU-1000 is weaker by 0.193 eV. In both environments, the sorption in the wide channels of NU-1000 is stronger than in the narrow channels. For UiO-67, MORPH adsorbs the strongest, and the B.O. is highest for “str. 1” geometry. NAL binds stronger for “str. 1” geometry, while the B.O. are similar among all structures. In all cases, the charge transfer is very low. At the same time, the bond orders are relatively high, indicating the dominant contribution of covalent and dispersion forces in the drug-MOF interactions, not the electrostatic ones.

Behavioral assays demonstrated that NU-1000 can mitigate morphine-induced locomotor hyperactivity and withdrawal symptoms. Furthermore, NU-1000 offers a promising avenue for enhancing the management of opioid dependence by the influence of locomotor activity and withdrawal symptoms associated with MORPH used in the in vivo studies.

The results presented in this paper are the culmination of the work within the project on the development of novel strategies based on metal–organic frameworks for the removal of drugs of abuse. Due to the global “opioid epidemic,” the results presented in this study open a new direction in materials-assisted medicine. Our preclinical studies clearly show an undeniable potential and safety for the living organisms of Zr-based MOFs in the emergency acute overdose treatment. Despite the promising results, further research is essential to assess the long-term efficacy and safety of Zr-MOFs in living organisms. Moreover, clinical trials focused on the application of Zr-MOFs as efficient therapeutic agents for adsorption and drug release could represent a breakthrough in their successful implementation in medical therapy on multiple levels.

## Supplementary Material









## Data Availability

The data that support the findings of this study are openly available in Hyjek, Kornelia; Dymek, Klaudia; Kurowski, Grzegorz; Boguszewska-Czubara, Anna; Budzyńska, Barbara; Navarro, Jorge A. Rodriguez; Borrego-Marin, Emilio; Mrozek, Weronika; Grymuza, Justyna; Pajdak, Anna; Piskorz, Witold; Śliwa, Paweł; Wielgosz, Alicja; Stachniuk, Anna; Fornal, Emilia; Jodłowski, Przemysław (2025), “Reviving the Fight Against Opioid Overdoses: Unleashing the Power of Metal-Organic Frameworks for Morphine Removal”, Mendeley Data, V1, doi: 10.17632/5ddv4gbh7p.1.

## Notes

Detailed synthesis procedures of MOFs; characterization details; literature survey on MORPH removal efficiency of common adsorbents, SEM microphotographs, DRIFT spectra; additional experimental results (PDF)
MORPH@NU-1000 (Movie S1) (MOV)
NAL@NU-1000 (Movie S2) (MOV)
MORPH, NAL@NU-1000 (Movie S3) (MOV)
