# Supplementary material for: Reviving the Fight against Opioid Overdoses: Unleashing the Power of Metal–Organic Frameworks for Morphine Removal
Source: ACS Appl Mater Interfaces. 2025 Nov 30;17(50):67670–89. doi: 10.1021/acsami.5c19297 (PMC12723641; doi:10.1021/acsami.5c19297)
Supplement: Supplementary file 1 [file am5c19297_si_001.pdf]

Supporting Information

Reviving the Fight Against Opioid Overdoses: Unleashing the Power of Metal-Organic Frameworks for morphine Removal

Kornelia Hyjek,[†] Klaudia Dymek,[†,@] Grzegorz Kurowski,[†] Anna Boguszewska-Czubara,[‡] Barbara Budzyńska,[¶] Jorge A. R. Navarro,[§] Emilio Borrego-Marin,[§] Weronika Mrozek,[¶] Justyna Grymuza,[‡] Anna Pajdak,[‖] Witold Piskorz,[⊥] Pawel Śliwa,[†] Alicja Wielgosz,[#] Anna Stachniuk,[#] Emilia Fornal,[#] and Przemysław J. Jodłowski[*,†]

†Faculty of Chemical Engineering and Technology, Cracow University of Technology, Warszawska 24, 31-155 Kraków, Poland

‡Department of Medical Chemistry, Medical University of Lublin, Chodźki 4A, 20-093 Lublin, Poland

¶Independent Laboratory of Behavioral Studies, Medical University of Lublin, Chodźki 4A, 20-093 Lublin, Poland

§Departamento de Química Inorgánica, Universidad de Granada, Granada 18071, Spain

‖Strata Mechanics Research Institute, Polish Academy of Sciences, Reymonta 27, 30-059 Kraków, Poland

⊥Faculty of Chemistry, Jagiellonian University in Kraków, Gronostajowa 2, 30-387 Kraków, Poland

#Department of Bioanalytics, Medical University of Lublin, Jaczewskiego 8b, 20-090 Lublin, Poland

@Lukasiewicz Research Network – Krakow Institute of Technology, Zakopiańska 73, 30-418 Kraków, Poland

Corresponding author: przemyslaw.jodlowski@pk.edu.pl

S1. Materials

Zirconium chloride ($ZrCl_4$, Merck, 98%), terephthalic acid (H_2BDC, Sigma Aldrich, 98%), 1,1'-biphenyl-4,4'-dicarboxylic acid (BPDC, Angene, 95%), zirconium oxide dichloride octahydrate ($ZrOCl_2 \times 8H_2O$, Alfa Aesar, 98%), benzoic acid (Acros, 99%), *N*,*N*-dimethylformamide (DMF, Chempur, Poland 99.8%), hydrochloric acid (Eurochem, 35-38%), methanol (CH_3OH, Chempur, 99%), morphine hydrochloride (Pharma-Cosmetic, Warsaw, Poland), naloxone hydrochloride (Sigma-Aldrich, Burlington, MA, USA), dioxne (Acros, 99%), tetrabromopyrene (Sigma Aldrich, 98%), (4-(methoxycarbonyl)phenyl)boronic acid (AmBeed, 98%), potassium phosphate tribasic (Sigma Aldrich, 98%), tetrakis(triphenylphosphine)-palladium(0) (Sigma, Aldrich, 99%), chloroform, (Acros, 98%) potassium hydroxide (KOH, Sigma Aldrich 90%), acetone (Chempur, 98%), sodium chloride (NaCl, Stanlab, p.a.), potassium chloride (KCl, Stanlab, p.a.), dipotassium hydrogen phosphate (K_2HPO_4, Chempur, p.a.), magnesium chloride hexahydrate ($MgCl_2 \times 6H_2O$, Chempur, p.), calcium chloride ($CaCl_2$, Chempur, p.a.), sodium sulfate anhydrous (Na_2SO_4, Chempur, p.a.), TRIS (Acros, 99%), and sodium hydrogen carbonate ($NaHCO_3$, Chempur, p.a.). The deionized water used was from a distiller and had a conductivity of 0.5 μS/cm. All chemicals were reagent grade and used without purification.

S2. Instrumentation

PXRD. The PXRD method determined the crystallinity of the synthesized MOFs, MORPH@MOF and NAL@MOF composites using an X'Pert Pro MPD diffractometer (PANalytical) with a copper lamp (CuKα radiation). The apparatus runs at 30 mA and 40 kV. Diffraction measurements were carried out in the 2θ range from 4° to 40° with a step of 0.033°.

DRIFTS. Infrared studies were made with a Diffuse Reflectance Infrared Fourier Transform Spectroscopy (DRIFTS) attachment, which enabled the study of functional groups and formed bonds on the surface of the materials. The apparatus was a Thermo is10 spectrometer equipped with a MCT detector. The measurement was carried out in an argon flow of 50 cm^3/min, so the sample was continuously degassed. In addition, before measurement, the sample was heated for 1 h at 110 ℃, providing dehydrated conditions. Spectra were taken in the range of 4000-650 cm^{-1} averaging 128 scans with 4 cm^{-1} resolution. A study was conducted for pristine MOFs, NAL@MOFs composites and composites after sorption MORPH@MOF.

SEM microimages were collected using an Apreo 2 S LoVac scanning electron microscope (Thermo Fisher Scientific). The instrument includes an electron beam deacceleration function and a backscattered electron (BSE) filter. In addition, it is equipped with a Schottky field emission scanning electron microscope (FESEM). Measurements are made in the low or high vacuum range from 1 pA to 50 nA and at an overhead voltage of 200 V to 30 kV. SEM images were taken at magnifications of 40× - 1,000,000×.

Low-temperature nitrogen adsorption. To determine the surface structure of pristine MOFs, NAL@MOF composites and MOFs after the MORPH sorption process, the volumetric low-temperature N_2 adsorption (LPNA) was used. The instrument operates in the 0-0.1 MPa pressure range and is called the ASAP 2020 analyzer. Firstly, the samples were prepared by degassing for 12 h under the pressure of 10^{-4} Pa and a temperature of 393 K. During the test isothermal conditions, a temperature of 77 K was maintained. Nitrogen was used as a gaseous adsorbate. The analysis made it possible to measure the surface area of the multilayer by the BET method S_{BET}, the single layer by the Langmuir method $S_{L,}$ and the pore volume. The last parameter was determined by the non-local density functional theory (NLDFT) method, designed for cylindrical pores and N_2 adsorbate (V_{DFT}).

HPLC. The method was utilized to evaluate the sorption kinetics of MORPH by MOFs: UiO-66, UiO-67, and NU-1000, using a Thermo Vanquish Core HPLC system equipped with Vanquish DAD CG, Vanquish Fluorescence Detector F, Vanquish Split Sampler CT and Column Compartment C. Separation was performed on an Accucore C18 150×3.0 mm^2, 2.6 µm column. Methanol CH_3OH (A) and 0.05% acetic acid CH_3COOH (B) were utilized as the mobile phase. The measurement lasted 10 min and was carried out in an isocratic manner of 5% A and 95% B at a flow rate of 0.5 mL/min. The injection volume of the samples was 25 µL. The spectrum was collected with a fluorescence detector using an excitation wavelength of λ = 280/335 nm. The MORPH retention time is approximately 2.2 min. The method determined the amount of adsorbed MORPH by MOFs in water and a solution simulating human body fluid SBF. Additionally, HPLC was used to determine the NAL release kinetics from: NAL@UiO-66, NAL@UiO-67 and NAL@NU-1000. The mobile phase was a mixture of eluents: 0.25 mM ammonium formate in 0.1% HCOOH (eluent A) and methanol CH_3OH (eluent B). Column was the same as an in the MORPH sorption experiment. The measurement was conducted in gradient flow for 20 min. At first, from 0 min to 1 min, the content of eluent B was 5%, while that of eluent A was 95%. Then, by 8 min of analysis, the content of eluent B increases to 95%, while that of eluent A decreases to 5%. This ratio is maintained until 12

min, after which there is a very rapid decrease in eluent B to 5% and an increase in eluent A to 95%, which occurs until 12.20 min of measurement. Analysis from 12.20 min to 20 min is carried out at a volume ratio of eluent B 5% and eluent A 95%. The volumetric flow rate was 0.3 cm^3/min, and the injection volume was 25 μL. The column was thermostated at 30 °C. The NAL retention time was 7.6 min at λ = 280 nm. The retention time for NAL in common experiment was 7.8 min and for MORPH was 4.4 min.

S3. Synthesis procedures of MOFs

UiO-66, and UiO-67 were synthesized just as in our previous studies described eslewhere[1]. Specific details of the synthesis of each MOFs are given below.

UiO-66 was prepared with $ZrCl_4$ as the metal part and benzene-1,4-dicarboxylic acids (H_2BDC) as ligands. In a beaker, 12.5 mL of DMF was measured and then 0.37 g (0.16 mmol) of $ZrCl_4$ was dissolved. Then 12.5 mL of hydrochloric acid HCl was added. Simultaneously, in a second beaker 0.26 g (1.56 mmol) of benzene-1,4-dicarboxylic acid (H_2BDC) was dissolved in 25 mL of DMF. The solutions were combined, and the whole was placed in Teflon cups, and autoclaved in turn. The solvothermal synthesis was carried out in an oven at 120 ℃ for 24 h. After this time, the solution was centrifuged at 6000 rpm for a time of 10 min and washed with DMF (1 × 25 mL) and CH_3OH (2 × 25 mL). The resulting precipitate was soaked with 25 mL of CH_3OH and left to stir at room temperature for 12 h. After this time, the MOF was centrifuged. It was activated in two stages: first in an oven under vacuum for 12 h at 120 ℃, then in an oven on air at 270 ℃ for 24 h.

UiO-67. 0.32 g $ZrCl_4$ (1.37 mmol) and 0.32 g 1,1'-biphenyl-4,4'-dicarboxylic acid (BPDC) (1.92 mmol) were dissolved in a mixture of 60 cm^3 DMF and 1.2 cm^3 trifluoroacetic acid. Then the mixture is placed in an ultrasonic bath for 10 min and then transferred in an autoclave. Synthesis is performed in an oven at 120 °C for 24 h. After synthesis, the material is washed with DMF - 20 cm^3 × 5 and acetone - 20 cm^3 × 3 using a centrifuge - 10 min, 6000 rpm. Activation of the material is carried out in a vacuum oven at 80 °C for 12 h.

NU-1000 material was synthesized following the protocol reported by T.C. Wang et al.[2] on a 1 g MOF scale.

Before the sorption studies, the MOFs were activated under vacuum at 120 ℃ for 12 h for UiO-66 and NU-1000 and 80 ℃ for 12 h for UiO-67.

S4. Results

Table S1. Literature survey on MORPH removal efficiency of common adsorbents

	Material	Results	References
1	Activated carbon	99.84% of morphine was absorbed from wastewater within 8 hours	(Gao et al., 2018)[3]
2	Activated carbon	Recoveries of 41.3% for morphine were obtained from assays performed in aqueous media spiked at the 30.0 mg L^{-1} level.	(Gonçalves et al., 2012)[4]
3	Activated carbon	41.8 % of morphine was absorbed from wastewater within 30 hours (10 mg L^{-1})	(Ling et al., 2017)[5]
4	Porous β-Cyclodextrin Polymers	98.2% of morphine was absorbed from wastewater within 30 hours (50 mg L^{-1})	(Ling et al., 2017)[5]
5	Ferric salts dosed to sewages (FeS)	97% of morphine was removed from wastewater within 6 hours (0.15−2 μg L^{-1})	(Kulandaivelu et al., 2019)[6]

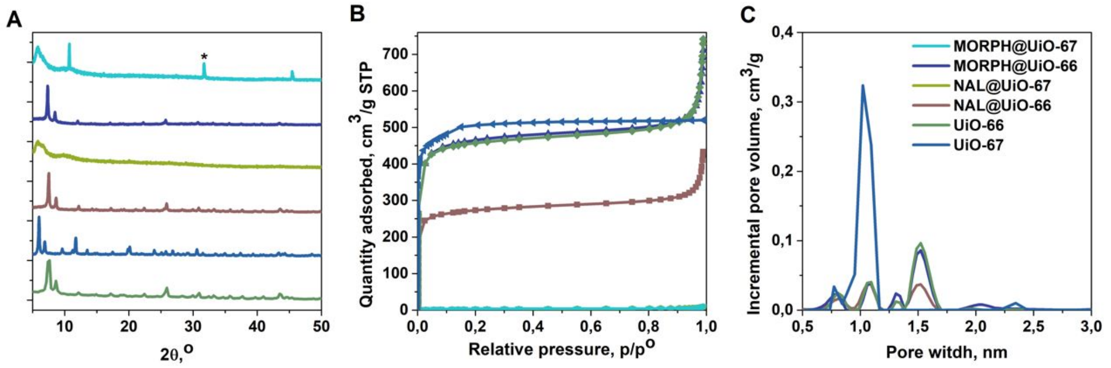

Figure S1 Characterisation results of pristine MOF (UiO-66, UiO-67), NAL@MOF and MORPH@MOF composites; (**A**) PXRD; (**B**) N_2 adsorption isotherms; (**C**) NLDFT pore size distribution.

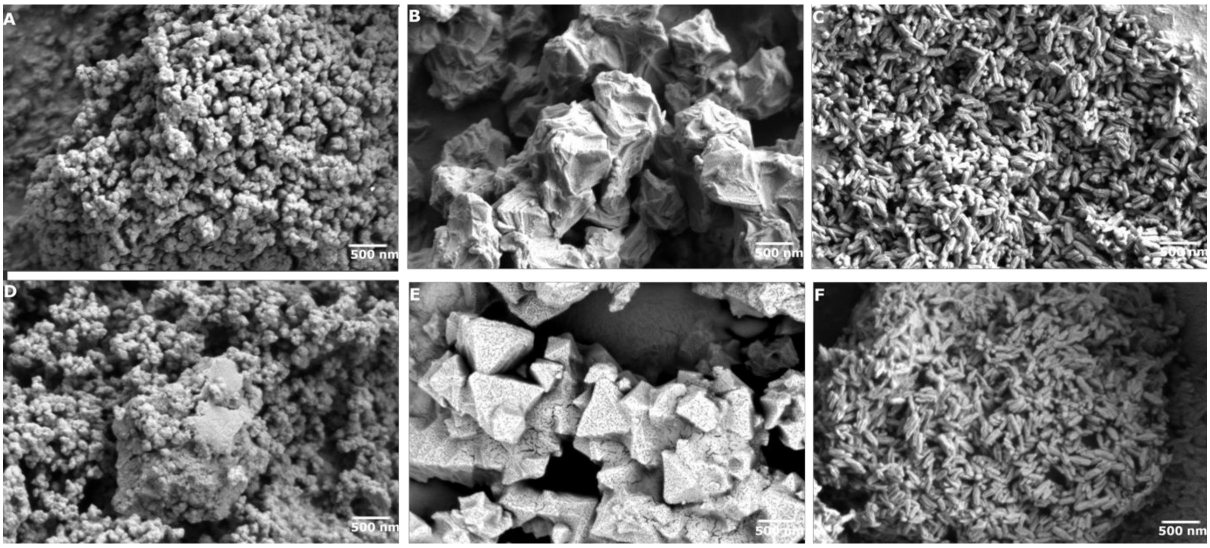

Figure S2 SEM microphotographs of MOFs after MORPH sorption process and NAL loaded; (**A**) MORPH@UiO-66; (**B**) MORPH@UiO-67; (**C**) MORPH@NU-1000; (**D**) NAL@UiO-66; (**E**) NAL@UiO-67; (**F)** NAL@NU-1000.

SEM analysis allowed us to understand the morphology MORPH@MOF and NAL@MOF composites (Figure S2). The differences in both the size and topology of the crystals can be seen between prepared samples. This is particularly evident for NU-1000 and UiO-67. The former composite is characterized by rod-shaped crystals of nanometre size. MORPH@UiO-67 has larger crystals with a diameter of about 1 μm and a more geometric shape. In the case of the MORPH@UiO-66 composite, the addition of hydrochloric acid during the MOF synthesis may have influenced the change in the size of the crystals. Microphotographs for NAL@MOF composites show very similar features to those for MORPH@MOF. The nanometric size is characterized by composites based on the UiO-66 and NU-1000 frameworks. Contrarily, in the case of NAL@NU-1000 and MORPH@NU-1000, the materials are in the form of small rods with sharp shapes. For NAL@UiO-66 and MORPH@UiO-66, the nanosized crystals are retained, but the shape is more spherical. For MORPH@UiO-66, the bulk has a more homogeneous character. Composites based on the UiO-67 structure are characterized by a micrometer size, of around 1 μm. The MORPH@UiO-67 material has larger grains, and a surface deposit is visible. This may indicate the presence of MORPH adsorbed on the material. The NAL@UiO-67 composite does not have a deposit such as for MORPH@UiO-67, however, and the particles although micrometric in size appear to be smaller.

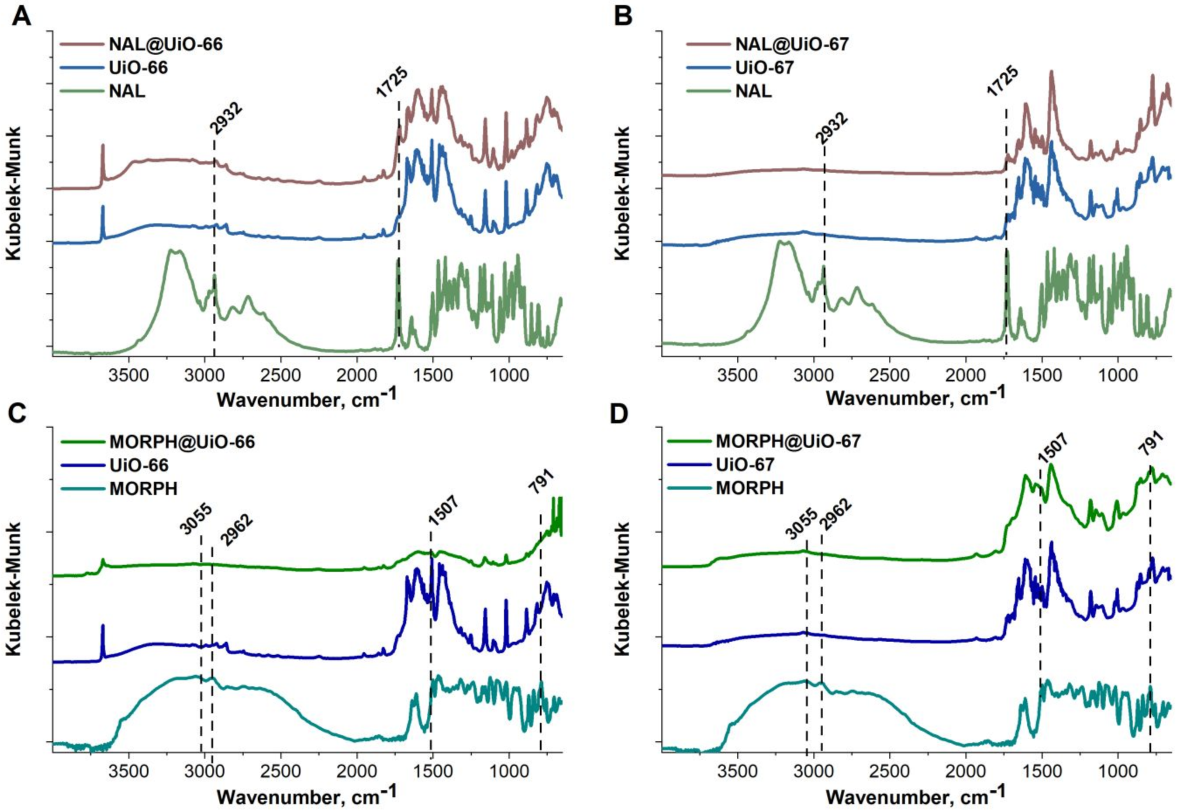

Figure S3 DRIFT spectra of prepared composites; (**A**) NAL@UiO-66; (**B**) NAL@UiO-67; (**C**) MORPH@UiO-66; (**D**) MORPH@UiO-67.

By analysing the MORPH spectrum and referring to literature data, it is possible to identify several characteristic bands[7,8,9]. For example, the band at 791 cm^{-1} corresponds to out-of-phase C-H bending vibrations. In addition, a band at 1018 cm^{-1} corresponds to ring torsion vibrations. MORPH is also characterized by a band at 1507 cm^{-1}, which is identified with C-C stretching vibrations. Two additional bands are at 2962 cm^{-1} and 3055 cm^{-1}. The former is responsible for the CH_3 asymmetric stretching vibration, and the latter for the C-H stretching vibration[9,10]. The spectrum of MORPH@NU-1000 contains the same bands we observe in MORPH@UiO-66, with two additional bands: 791 cm^{-1} and 2956 cm^{-1}, the latter of which may correspond to the stretching asymmetric vibrations of CH_3. This band is slightly shifted from the spectrum of pure MORPH, where it occurs at 2962 cm^{-1}, as described in main article.

Typical bands on the DRIFT spectrum characterizing NAL are also two bands: one at 1604 cm^{-1} and the other at 1725 cm^{-1}, corresponding to C=C and C=O vibration, respectively[11]. The band relating to the N-H group occurring at 2932 cm^{-1} is also evident, while the band occurring

at 3421 cm^{-1} comes from the -OH group of adsorbed water. Performing a comparative analysis between the spectra of pristine MOFs, the NAL molecule, and the NAL@MOF composites, significant differences are evident. More specifically, on the spectra of the composites it is possible to notice the presence of bands occurring on the spectrum of pure NAL and absent on the spectra of pure MOFs.

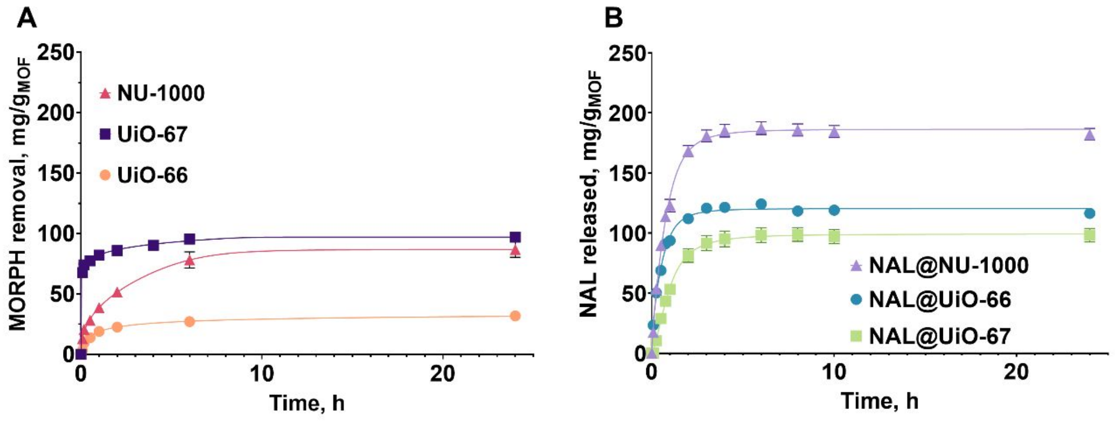

Figure S4 (**A**) MORPH sorption and (**B**) NAL release by MOFs in H_2O.

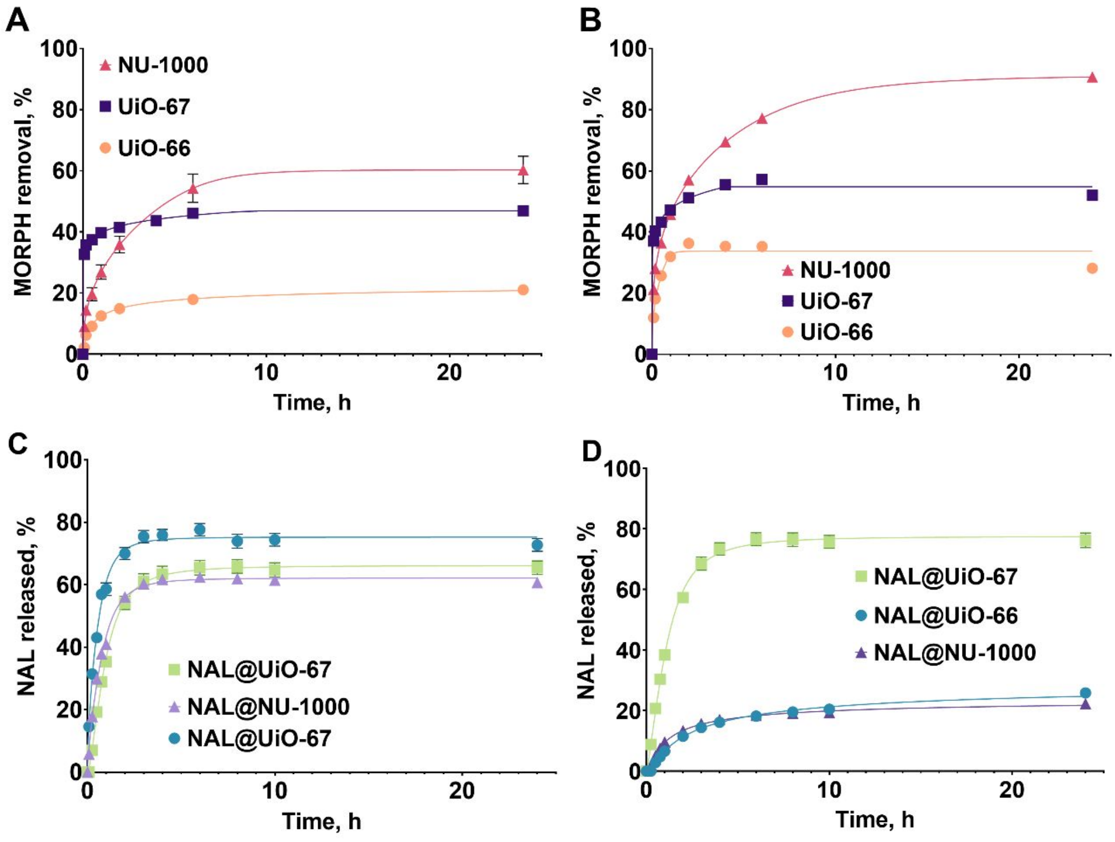

Figure S5 (**A, B**) MORPH sorption and (**C, D**) NAL release in H_2O **(A, C)** and SBF **(B, D)**.

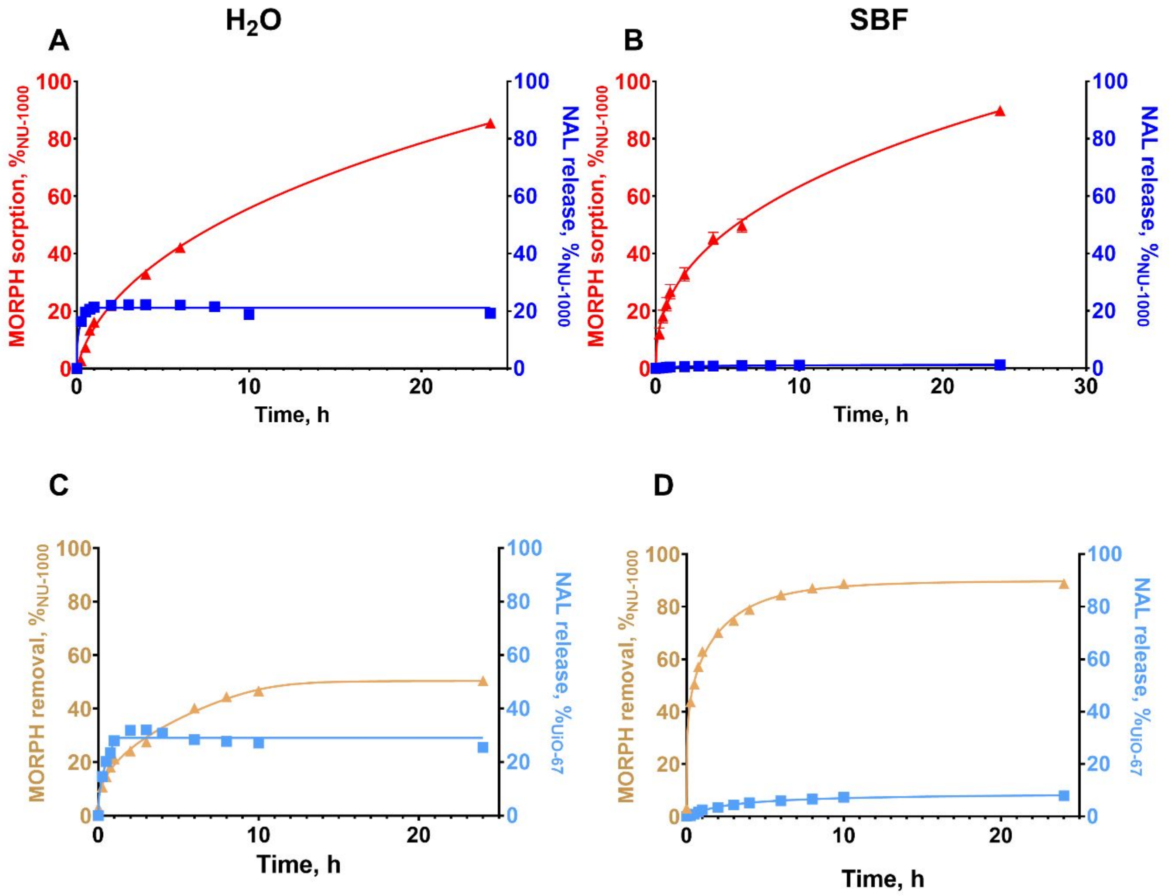

Figure S6 Simultaneous MORPH sorption experiment and NAL release from NU-1000 **(A-B)** and mixture of NU-1000 (MORPH adsorption) and UiO-67 (NAL release) **(C-D)** in **(left)** H_2O; **(right)** SBF environment.

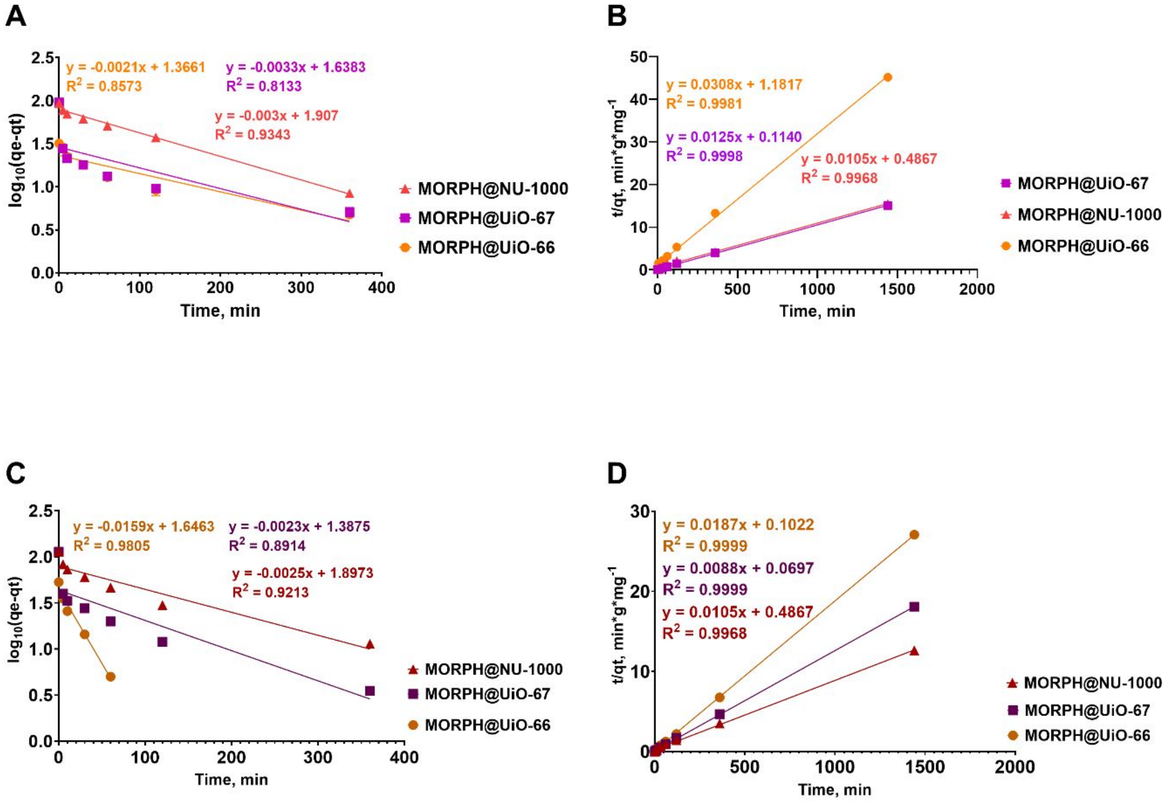

Figure S7 Pseudo-first-order (**A**, **C**) and pseudo-second-order (**B**, **D**) kinetics for MORPH sorption by Zr-MOFs in different media; (**A**, **B**) H_2O; (**C**, **D**) SBF.

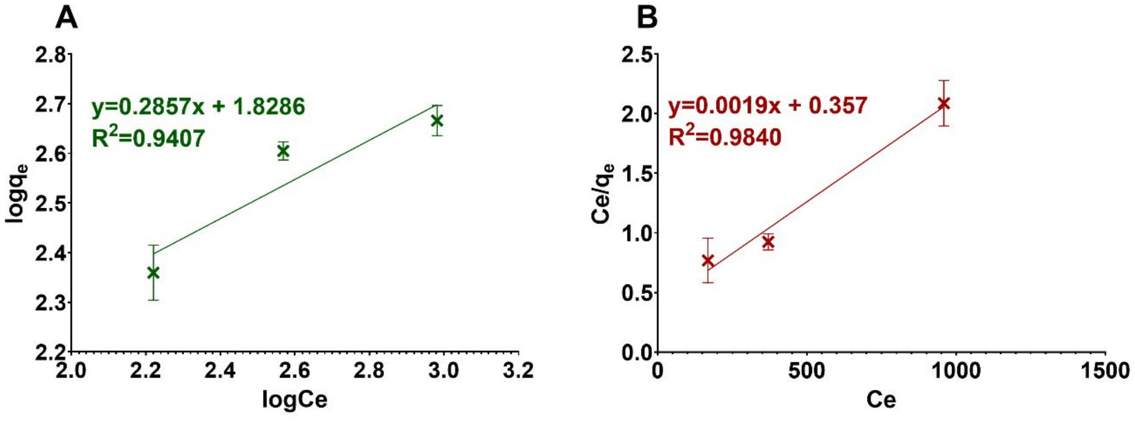

Figure S8 (**A**) Langmuir and (**B**) Freundlich isotherm for MORPH sorption by NU-1000.

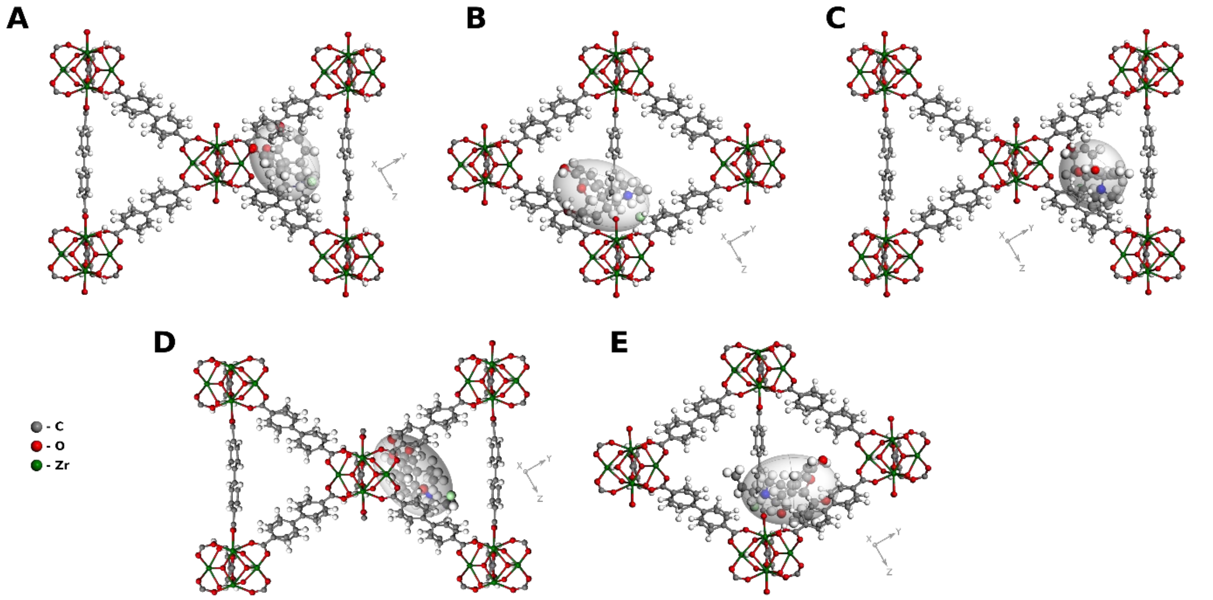

Figure S9 DFT optimized structures of MORPH-HCl adsorbed in UiO-67: (**A**) str.1, (**B**) str.2, (**C**) str.3; DFT optimized structures of NAL-HCl adsorbed in UiO-67: (**D**) str.1, (**E**) str.2.

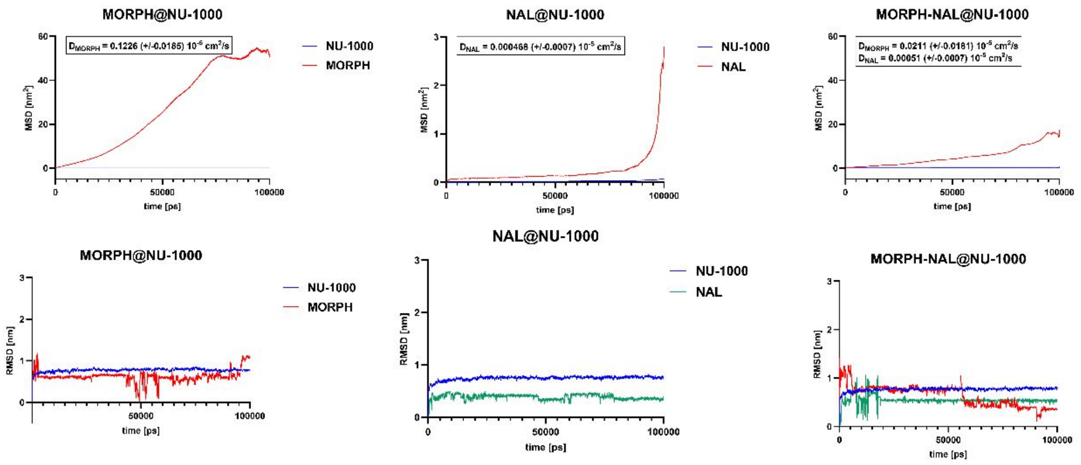

Figure S10 The mean square displacement (MSD) during the 100 ns MD simulations, which was calculated in three dimensions for MORPH@NU-1000, NAL@NU-1000, and MORPH-NAL@NU-1000, and the estimated diffusion coefficients. The lower graphs depict the evolution of RMSD throughout the simulation.

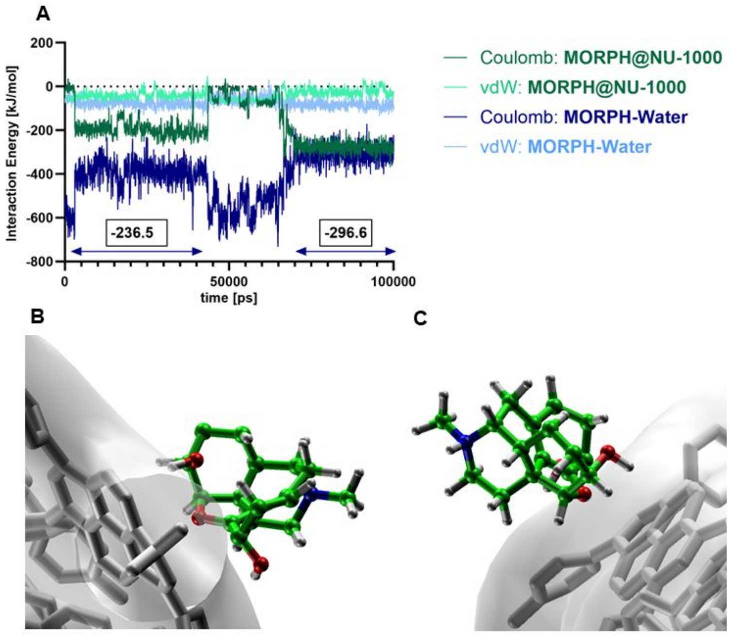

Figure S11 (A) Changes of interaction energies between the NU-1000 and MORPH along with MD simulation. Close-up views of the two possible complexes with MORPH **(B, C)** molecules in the channels of MOF. The structures are representative after hierarchical clustering based on RMSD; Water molecules and hydrogen atoms of MOF are invisible.

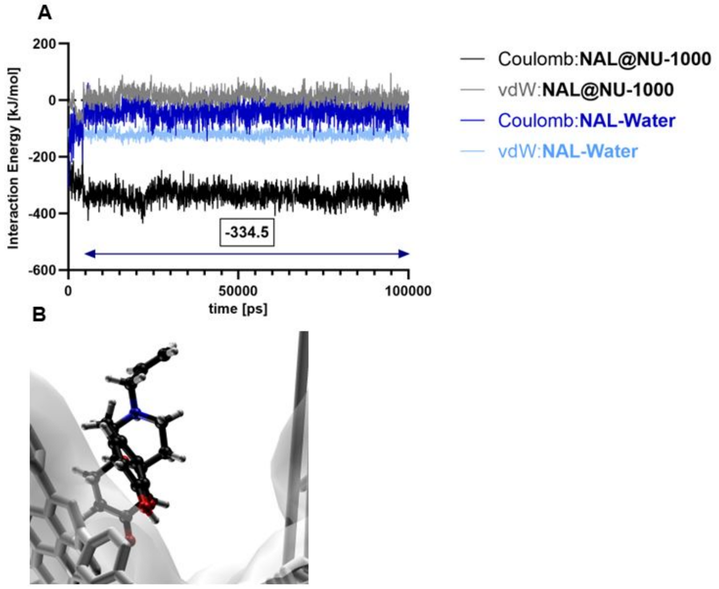

Figure S12 (**A**) Changes of interaction energies between the NU-1000 and NAL (NAL) along with MD simulation. Close-up views of the complex with NAL molecule (**B**) in the channels of MOF. The structure is representative after hierarchical clustering based on RMSD; Water molecules and hydrogen atoms of MOF are invisible.

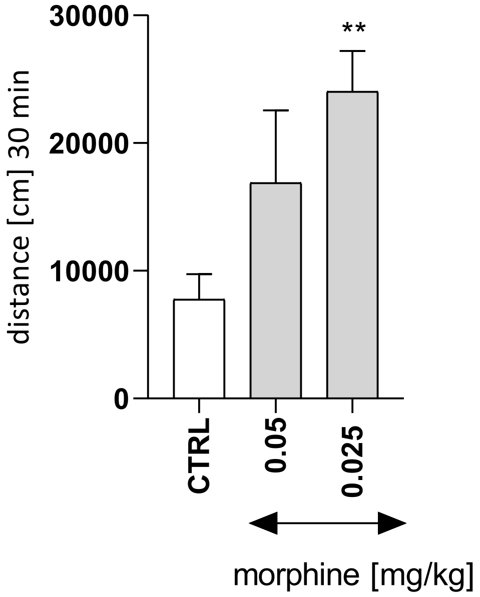

Figure S13 Effect of MORPH(0.025 and 0.05 mg/kg, iv.) on spontaneous locomotor activity in mice. Locomotor activity (number of interruptions of light beams) was recorded for the 30 min. Data is presented as the means ± SEM. n = 8; ** p < 0.01, vs. saline-treated (post hoc Tukey's test).

Physical Dependence and Effects of NAL@UiO-66 and NAL@UiO-67 on the Expression of MORPH Withdrawal Signs in Mice

Table S2. Effect of NAL@UIO-66 (NAL@UiO-66 30 mg/kg, i.p.) and NAL@UiO-67 (NAL@UiO-67 30 mg/kg,i.p.) on MORPH dependence in mice. MORPH was administered for eight consecutive days (10 mg/kg, 15 mg/kg, 20 mg/kg, 25 mg/kg, 30 mg/kg, 35 mg/kg, 40 mg/kg, and 50 mg/kg), twice a day. On the 9th day, MORPH (50.0 mg/kg) was first administered and 1 h later, NAL (2 mg/kg, i.p.) was injected. The animals were immediately placed in glass cylinders and number of MORPH jumping behaviors was recorded for 30 min. To study the effect of NAL@UiO-66 and NAL@UiO-67 on the expression of MORPH withdrawal signs, NAL@MOF was administered on day 9th, after MORPH injection, and the number of MORPH jumping behaviors was observed for 30 min. The results are shown as the average number of jumping behaviors ± SEM. ****p < 0.0001 vs. saline group, (Bonferroni's test).

	NAL	UiO66	NAL@UiO-66	UiO67	NAL@UiO-67
saline	0±0	0±0,0	0±0,0	0±0,0	0±0,0
MORPH	61,00± 14,99 ****	0±0,0	0±0,0	0±0,0	0±0,0

Two-way ANOVA revealed significant differences in the studied mice (MORPH effect: F F (1, 77) = 38,72, *p*<0,0001; MOFs effect F (1, 77) = 38,72, *p*<0,0001; interaction: F (5, 77=33,77, P<0,0001). Only the administration of NAL in mice chronically treated with MORPH induced a marked increase (*p* < 0.0001) in the number of jumping behaviors compared to MORPH-treated mice. The administration of NAL@MOFs (UiO-66: 30 mg/kg, UiO-67: 30 mg/kg, i.p.) did not influence the jumping behaviors compared to the MORPH group. NAL@MOFs alone did not produce any jumping behavior in mice treated with either saline or saline with NAL.

Bibliography

(1) Hyjek, K.; Kurowski, G.; Dymek, K.; Boguszewska-Czubara, A.; Budzyńska, B.; Wronikowska-Denysiuk, O.; Gajda, A.; Piskorz, W.; Śliwa, P.; Szumera, M.; Jeleń, P.; Sitarz, M.; Jodłowski, P. J. Metal-Organic Frameworks for Efficient Mephedrone Detoxification or Supervised Withdrawal – Synthesis, Characterisation, and in Vivo Studies. *Chem. Eng. J.* **2024**, *479* (July 2023), 147655. https://doi.org/10.1016/j.cej.2023.147655.

(2) Wang, T. C.; Vermeulen, N. A.; Kim, I. S.; Martinson, A. B. F.; Fraser Stoddart, J.; Hupp, J. T.; Farha, O. K. Scalable Synthesis and Post-Modification of a Mesoporous Metal-Organic Framework Called NU-1000. *Nat. Protoc.* **2016**, *11* (1), 149–162. https://doi.org/10.1038/nprot.2016.001.

(3) Gao, X.; Bakshi, P.; Sunkara Ganti, S.; Manian, M.; Korey, A.; Fowler, W.; Banga, A. K. Evaluation of an Activated Carbon-Based Deactivation System for the Disposal of Highly Abused Opioid Medications. *Drug Dev. Ind. Pharm.* **2018**, *44* (1), 125–134. https://doi.org/10.1080/03639045.2017.1386199.

(4) Goncalves, A. F. P.; Neng, N. R.; Mestre, A. S.; Carvalho, A. P.; Nogueira, J. M. F. Development of a Powdered Activated Carbon in Bar Adsorptive Micro-Extraction for the Analysis of Morphine and Codeine in Human Urine. *J. Chromatogr. Sci.* **2012**, *50* (7), 574–581. https://doi.org/10.1093/chromsci/bms051.

(5) Ling, Y.; Klemes, M. J.; Xiao, L.; Alsbaiee, A.; Dichtel, W. R.; Helbling, D. E. Benchmarking Micropollutant Removal by Activated Carbon and Porous β-Cyclodextrin Polymers under Environmentally Relevant Scenarios. *Environ. Sci. Technol.* **2017**, *51* (13), 7590–7598. https://doi.org/10.1021/acs.est.7b00906.

(6) Kulandaivelu, J.; Gao, J.; Song, Y.; Shrestha, S.; Li, X.; Li, J.; Doederer, K.; Keller, J.; Yuan, Z.; Mueller, J. F.; Jiang, G. Removal of Pharmaceuticals and Illicit Drugs from Wastewater Due to Ferric Dosing in Sewers. *Environ. Sci. Technol.* **2019**, *53* (11), 6245–6254. https://doi.org/10.1021/acs.est.8b07155.

(7) Petruševski, G.; Acevska, J.; Stefkov, G.; Poceva Panovska, A.; Micovski, I.; Petkovska, R.; Dimitrovska, A.; Ugarkovic, S. Characterization and Origin Differentiation of Morphine Derivatives by DSC/TG and FTIR Analysis Using Pattern Recognition Techniques. *J. Therm. Anal. Calorim.* **2016**, *123* (3), 2561–2571. https://doi.org/10.1007/s10973-016-5242-z.

(8) Baranska, M.; Kaczor, A. Morphine Studied by Vibrational Spectroscopy and DFT Calculations. *J. Raman Spectrosc.* **2012**, *43* (1), 102–107. https://doi.org/https://doi.org/10.1002/jrs.3005.

(9) Pandey, A. K.; Dwivedi, A.; Siddiqui, S. A.; Misra, N. Vibrational Spectra of Two Narcotics-A DFT Study. *Chinese J. Phys.* **2013**, *51* (3), 473–499. https://doi.org/10.6122/CJP.51.473.

(10) Misra, N.; Dwivedi, A.; Pandey, A. K.; Trivedi, S. Vibrational Analysis of Two Narcotic Compounds-Codeine and Morphine - a Comparative DFT Study. *Der Pharma Chem.* **2011**, *3* (3), 427–448.

(11) Chen, K.; Chang, H. H. R.; Lugtu-Pe, J.; Gao, Y.; Liu, F.-C.; Kane, A.; Wu, X. Y. Exploration of a Novel Terpolymer Nanoparticle System for the Prevention of Alcohol-Induced Dose Dumping. *Mol. Pharm.* **2024**, *21* (12), 6257–6269. https://doi.org/10.1021/acs.molpharmaceut.4c00706.
